# Contrasting Dispersal Patterns of Co‐Occurring Benthic Sister Sea Star *Labidiaster* Species (Asteroidea: Heliasteridae) in the Southern Ocean

**DOI:** 10.1002/ece3.73450

**Published:** 2026-04-10

**Authors:** Nicola Rodewald, Nerida G. Wilson, Sally C. Y. Lau, Jan M. Strugnell

**Affiliations:** ^1^ Centre for Sustainable Tropical Fisheries and Aquaculture James Cook University, College of Science and Engineering Townsville Queensland Australia; ^2^ Securing Antarctica's Environmental Future James Cook University Townsville Queensland Australia; ^3^ Scripps Institution of Oceanography University of California San Diego La Jolla California USA; ^4^ School of Biological Sciences University of Western Australia Perth Western Australia Australia; ^5^ Securing Antarctica's Environmental Future Western Australian Museum Welshpool Western Australia Australia

**Keywords:** Antarctic Polar Front, genetic connectivity, population structure

## Abstract

Closely related and co‐occurring species offer valuable systems to assess whether dispersal barriers in shared environments are navigated in a consistent manner. *Labidiaster radiosus* and *L. annulatus* have been morphologically described as two separate species from southern South America and the Southern Ocean respectively, yet the phylogenetic relationships and boundaries between these species have still not been genetically clarified. *Labidiaster annulatus* has pelagic larvae; however, the developmental mode for *L. radiosus* has not been confirmed. This study aimed to employ genetic methods to analyze the phylogeny, distribution, species boundaries, population structure, demographic history and genetic connectivity for *L. radiosus* and *L. annulatus* . Two molecular markers were sequenced in this study including a part of the mitochondrial gene cytochrome c oxidase subunit I (COI) and the intron of the nuclear adenosine triphosphate synthetase, beta subunit gene (ATPSβ—intron 7). A sister‐species relationship was revealed between *L. radiosus* and *L. annulatus* ; both species displayed circum‐Antarctic distributions, extending their known distributions and were found to co‐occur at Heard, Balleny and Scott Islands. *Labidiaster radiosus* and *L. annulatus* were estimated to have diverged ~3.56 mya (HPD95: 5.25–2.15). Additionally, *L. radiosus* was confirmed to have pelagic larvae in the Southern Ocean. *Labidiaster radiosus* population structure revealed clear subclades north and south of the APF, which diverged ~2.08 mya (HPD95: 3.48–0.58). Analyses for *Labidiaster annulatus* indicated high genetic connectivity among all locations across the Southern Ocean. Historical dispersal analyses showed high genetic connectivity among Scotia Sea Islands, and the South Sandwich Islands were identified as a possible contemporary sink population. Despite their similarities in life history and circum‐Antarctic distribution, *L. radiosus* and *L. annulatus* revealed contrasting population structures. This highlights the unpredictable nature of dispersal for Southern Ocean benthic invertebrates when based on life history alone.

## Graphical abstract

This study aimed to employ genetic methods to analyze the phylogeny, species boundaries, population structure, demographic history and genetic connectivity for Southern Ocean sea stars, *Labidiaster radiosus* and *L. annulatus* . A sister‐species relationship was revealed between *L. radiosus* and *L. annulatus* ; both species displayed circum‐Antarctic distributions, extending their known distributions. Despite their similarities in life history and circum‐Antarctic distribution, *L. radiosus* and *L. annulatus* revealed contrasting population structures. This highlights the unpredictable nature of dispersal for Southern Ocean benthic invertebrates when based on life history alone.

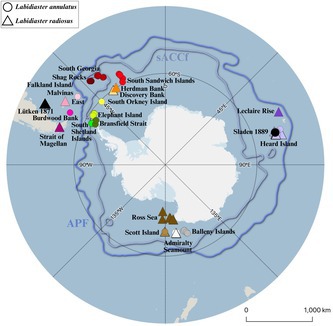

## 1 Introduction

Understanding how benthic invertebrates disperse can offer valuable insights into population connectivity. Dispersal occurs when an individual moves across space, leaving a signature of gene flow among populations (Burgess et al. 2016; Greenwood 1980; Greenwood and Harvey 1982; Howard 1960; Newton and Brockie 2003; Ronce 2007). Historically, the existence of a pelagic developmental phase in benthic invertebrates would suggest a high dispersal potential across a wide geographical range (Haye et al. 2014, 2012; Lester et al. 2007). Conversely, benthic invertebrates lacking pelagic developmental modes (direct‐developers) were assumed to have a low dispersal potential and, therefore, would not be expected to disperse across large geographical distances (Ayre et al. 2009; Bohonak 1999; Haye et al. 2014; Hedgecock 1986). It is now known that long‐distance and high dispersal potential is possible for some benthic invertebrates, regardless of their life history (e.g., Cumming et al. 2014; Güller et al. 2020; Linse et al. 2007; Salloum et al. 2020). However, quantifying dispersal directly can be challenging in marine environments due to logistical difficulties in directly observing the dispersal movement (Abesamis et al. 2016) of benthic invertebrates; therefore, genetic approaches such as assessing genetic connectivity among populations are valuable for inferring dispersal patterns (e.g., González‐Wevar et al. 2023; Lau et al. 2024; Lau, Strugnell, et al. 2023). Genetic approaches can provide powerful tools for investigating dispersal in samples collected from remote and hard‐to‐reach environments, such as the Southern Ocean, where direct observation of dispersal is exceptionally challenging.

The Antarctic Polar Front (APF) has been recognized as a biogeographical barrier separating sub‐Antarctic waters to the north from Antarctic waters to the south. The physical environmental changes along this biogeographical barrier can hinder dispersal across the APF. The APF has been identified as a convergence zone categorized by an increase in eddies (moving south to north) and an increase in temperature (south to north) (see Cotroneo et al. 2013). The APF passes through the Drake Passage, which reaches depths up to ~4.2 km in some locations, creating additional barriers to benthic dispersal (see Oldenhage et al. 2024; figure 1 in Riley et al. 2019). The Drake Passage was formed when the South American and Antarctic continents split apart around 50 to 17 million years ago (still debated) (see Barker et al. 2007; Dalziel et al. 2013; Hodel et al. 2021; Pérez et al. 2019; Scher and Martin 2006; Van De Lagemaat et al. 2021). The formation of the Drake Passage allowed the circum‐Antarctic flow of the Antarctic Circumpolar Current (ACC) (see Hodel et al. 2021). The modern ACC was intensified ~14 million years ago (see Evangelinos et al. 2024) and reinforces the APF through strong westerly currents that can impede north–south dispersal. However, dispersal across the APF is possible for some benthic invertebrates (see Dietz et al. 2019; Galaska et al. 2017; Moles et al. 2021; Moore et al. 2018), but this requires benthic invertebrates to overcome extreme distances, depths, temperature changes and strong currents (e.g., Hunter and Halanych 2008; Shaw et al. 2004).

The Scotia Sea, located adjacent to the Drake Passage and south of the APF, may facilitate dispersal across the APF via a chain of habitable islands (Scotia Arc islands). The Scotia Arc islands have been suggested to aid the dispersal of a sponge, *Mycale* (*Oxymycale*) *acerate* Kirkpatrick, 1907 (Leiva et al. 2022), and the Antarctic fishes *Notothenia rossii* Richardson, 1844 and *Champsocephalus gunnari* Lönnberg, 1905 (Young et al. 2018). Similarly, the Scotia Arc islands have also been found to aid dispersal within the Scotia Sea for the benthic sea star *Glabraster antarctica* Smith, 1876 (Moore et al. 2018). Moore et al. (2018) also identified long‐distance dispersal among South American, Shag Rocks, Heard Island, and Ross Sea (Scott A) locations. Shag Rocks was genetically isolated from the rest of the Scotia Arc locations. However, the low genetic differentiation recovered between South American and Shag Rocks locations indicates that gene flow can occur across the APF, indicating that the APF can be a permeable barrier. Due to its wide Southern Ocean and southern South American distribution, the benthic sea star genus *Labidiaster* Lütken (1871) (Asteroidea: Heliasteridae) can be used to improve the understanding of dispersal patterns within the Scotia Arc region and across the APF.

The large, multi‐rayed sea star genus *Labidiaster* is comprised of two species: the South American *Labidiaster radiosus* Loven in Lütken (1871) and the Southern Ocean *Labidiaster annulatus* Sladen (1889). The genus *Labidiaster* can be morphologically distinguished from other genera in the family Heliasteridae by having a large number of rays and very large pedicellariae (Botto et al. 2006; Dearborn et al. 1991; Madsen 1956; Sladen 1889). Morphologically, *L. annulatus* may have more than 50 rays (Dearborn et al. 1991), and *L. radiosus* can have 23 to 42 rays (Perrier 1891, 67); however, it is hard to differentiate juvenile *Labidiaster* species as they have not yet developed their full complement of rays (Hotchkiss 2000; Perrier 1891). These two species are morphologically similar and can be distinguished in adult specimens, though identification remains challenging even for specialists. For example, *L. annulatus* can be identified by the variation of big and small spines on its disk and the presence of abactinal plates in arm arcs (Mutschke and Mah 2009). Both species can occur on a variety of bottom types, including sand, mud, gravel, and rocks, and are found between 23 and 1395 m in water depths (Dearborn et al. 1991; Janosik et al. 2008; this study in Tables A1 and A2). For a long period, *L. annulatus* was thought to be distributed only within the Southern Ocean, and *L. radiosus* was thought to be distributed around southern South America in the South Atlantic Ocean, including the Falkland Islands/Malvinas (see Table A1). More recently, specimens of *L. radiosus* have been identified within the Scotia Arc (Barboza et al. 2011), the Kerguelen Plateau, and possibly the Antarctic Peninsula (Vantomme et al. 2023), suggesting high dispersal potential may be possible for both species.

Pelagic developmental mode of benthic invertebrates has been used to predict high dispersal potential and explain a large geographical range (Haye et al. 2014, 2012; Lester et al. 2007). Janosik et al. (2008) identified three bipinnaria and one brachiolaria larva of *L. annulatus* from the western Antarctic Peninsula region using 16S (mitochondrial DNA) and inferred planktotrophic larval development for *L. annulatus* . *Labidiaster radiosus* was predicted to also produce planktotrophic larvae that enabled dispersal, like its sister‐species *L. annulatus* (Janosik et al. 2008). Instead of using the pelagic development mode for *L. annulatus* as a predictor to identify high dispersal potential, genetic methods can be used to help understand patterns of dispersal.

The overarching aim of this study is to employ genetic methods to provide insight into the dispersal of the Southern Ocean benthic invertebrates *L. radiosus* and *L. annulatus* . To inform this aim, this study will first identify phylogenetic relationships between *L. radiosus* and *L. annulatus* . Then, patterns of genetic diversity and population structure between and within *L. radiosus* and *L. annulatus* throughout their distribution range will be examined. Finally, patterns of genetic connectivity within the Scotia Arc region and across the APF will be investigated to identify potential dispersal barriers affecting *Labidiaster* species.

## 2 Materials and Methods

### 2.1 Sample Collection and DNA Extraction

Tissue was subsampled from 70% to 98% ethanol‐preserved *Labidiaster* (*n* = 325) specimens that were collected from previous Southern Ocean voyages. These were accessed from collections stored at the Western Australian Museum (WAM), National Institute of Water and Atmospheric Research (NIWA), Scripps Institution of Oceanography Benthic Invertebrates collection (SIO‐BIC), and Queensland Museum Tropics, Townsville (QMT) (Table A2).

The Qiagen DNeasy Blood and Tissue Kit was used to extract genomic DNA according to the manufacturer's instructions (Qiagen GmbH, Hilden, Germany). Quality and quantity checks were conducted using electrophoresis (0.8% agarose gel), spectrophotometry (Nanodrop, Thermo Fisher Scientific), and/or fluorometry (Quantus Fluorometer, Promega).

### 2.2 PCR and Sequencing

Two molecular markers were sequenced in this study. These include a partial fragment of the mitochondrial (mtDNA) gene cytochrome c oxidase subunit I (COI) and the intron of the nuclear (nuDNA) adenosine triphosphate synthetase, beta subunit gene (ATPSβ—intron 7). The mtDNA primers COIceF (ACTGCCCACGCCCTAGTAATGATATTTTTTATGGTNATGCC) and COIceR (TCGTGTGTCTACGTCCATTCCTACTGTRAACATRTG) were used (Geller et al. 2013). The nuDNA primers Int7F2 (GGGCTCCATCACCTCAGTACAGGT) and ATPS7R (CGGTCAGATCATCAGCTGGNACRTADAT) were used (Foltz, Bolton, et al. 2007). Genomic DNA was amplified via PCR in a 25 μL reaction. The reaction volume consisted of 12.5 μL of OneTaq Quick‐Load 2X Master Mix with Standard Buffer (New England Biolabs), 0.5 μL of forward primer (10 μM) and 0.5 μL of reverse primer (10 μM), and 1 μL of Bovine Serum Albumin. Molecular biology grade water and DNA template (concentrations ranged from 5 to 40 ng/μL) were added to comprise the 25 μL PCR reaction volume. The PCR cycle conditions for COI consisted of an initial denaturation for 3 min at 95°C, followed by 40 cycles of 45 s at 94°C, 1.10 min at 42°C–47°C, 1.20 min at 72°C, and a final extension of 5 min at 72°C (Hoareau and Boissin 2010). The PCR cycle conditions for the ATPSβ—intron 7 started with an initial denaturation of 2 min at 94°C, followed by 35 cycles of 20 s at 94°C, 1 min at 42°C–45°C, 1 min at 72°C and a final extension for 6 min at 72°C (Jarman et al. 2002). The PCR products were visually inspected using electrophoresis on a 1.5% agarose gel with a HyperLadder IV 100 bp (Bioline) under ultraviolet light. Unpurified PCR products were sent to the Australian Genome Research Facility (Brisbane) for amplicon purification and dual‐direction Sanger sequencing using BigDye Terminator version 3.1 chemistry (Applied Biosystems) with Applied Biosystems 3730 and 3730xl DNA Analyzers, under standard cycling PCR conditions.

### 2.3 Data Analyses

Consensus sequences were built from forward and reverse reads from the same individual. Poor‐quality or incorrectly called bases were edited or trimmed. DNA sequences were aligned using default settings with the MAFFT plugin (Katoh and Standley 2013) in Geneious Prime 2024.0.5 (https://www.geneious.com). For species identification, all sequences were examined against existing sequences using the MegaBLAST server within the NCBI databases (Altschul et al. 1990; Morgulis et al. 2008).

Intron 7 nuDNA regions are known to contain heterozygote sites (Foltz 2007). Heterozygotes were confirmed by manual inspection of bidirectional traces in Geneious Prime and the Find Heterozygotes plugin. To distinguish between true heterozygous sites and ambiguous sites (i.e., potential sequencing error), the intron 7 sequences were phased using PHASE version 2.1 (Stephens et al. 2001; Stephens and Donnelly 2003), implemented through DnaSp version 6 (Rozas et al. 2017), with default settings. Phasing separated the genetic information of an individual into two distinct sequences; thus, each sequence represented an allele inherited from one parent (Raj et al. 2024; Schanzer et al. 2020).

#### 2.3.1 Phylogenetic Reconstruction and Species Delimitation

The COI and the intron 7 datasets were analyzed separately. Phylogenetic trees were constructed using maximum likelihood and Bayesian inference to determine the evolutionary relationships among sequences. The best‐fit models for all phylogenetic trees were obtained using ModelFinder (Kalyaanamoorthy et al. 2017), which was run on the IQ‐TREE web server (http://iqtree.cibiv.univie.ac.at/ last accessed in June 2024; Trifinopoulos et al. 2016). The Bayesian information criterion (BIC) identified the best‐fit model for the COI dataset as HKY + F + G4 and for the intron 7 dataset as HKY + F + I. Maximum likelihood trees were constructed using the IQ‐TREE web server with 1000 ultrafast bootstrap replicates, and the remaining parameters were set to default (Hoang et al. 2018; Nguyen et al. 2015). The Bayesian inference trees were constructed in Geneious Prime using the MrBayes plugin (Huelsenbeck and Ronquist 2001; Ronquist et al. 2012). Four parallel Markov chains were run for 10 million generations, sampling every 1000 generations, and the first 25% of trees were excluded as burn‐in (Zhang et al. 2024). Forty million MCMC post‐burn‐in generations using Tracer version 1.7.2 (Rambaut et al. 2018) were reviewed for convergence, and effective sample sizes (ESS) were ensured to be greater than 200. Maximum likelihood and Bayesian inference trees were viewed using FigTree version 1.4.3 (Rambaut et al. 2016).

The BLASTN analysis for all COI sequences revealed a high percentage identity (> 99%) score (Altschul et al. 1990) with an unidentified *Asteriidae* sp. bipinnaria larvae (Mary Sewell, pers. comm.) sequence (GenBank: GU227094, Figure S1) from the Ross Sea (Heimeier et al. 2010). Therefore, GU227094 was included in the COI phylogenetic trees, which were rooted using a sequence (GenBank: JX130054) from the outgroup species *Heliaster helianthus* Lamarck, 1816 (family Heliasteridae Viguier, 1878). The phylogenetic trees for the intron 7 dataset were rooted at midpoint as no appropriate outgroup sequences were available.

Species delimitation was executed using likelihood analyses in the Automatic Barcode Gap Discovery (ABGD) web interface (https://bioinfo.mnhn.fr/abi/public/abgd/ last accessed 13 June 2024) and Assemble Species by Automatic Partitioning (ASAP) web interface (https://bioinfo.mnhn.fr/abi/public/asap/asapweb.html last accessed 13 June 2024) (Puillandre et al. 2021, 2012). The IQ‐TREE selected (GTR + F + G4) substitution model for the COI dataset was not available in ABGD and ASAP. Therefore, a distance matrix was generated using IQ‐TREE, corrected by the GTR + F + G4 substitution model, which was employed for both the ABGD and ASAP analyses (see Ranasinghe et al. 2022). Two initial partitions for the intraspecific distance represented by 0.001 < *p* > 0.01 were used from the ABGD output to identify the maximum number of species (e.g., Maroni et al. 2022; following Puillandre et al. 2012). The maximum number of species was defined by the two lowest ASAP‐scores of 1.00 and 2.00 (ASAP 1st and 2nd, respectively; see Maroni et al. 2022; following Ranasinghe et al. 2022; Solovyeva et al. 2023). Similar to ABGD, the ASAP multipartition approach was used to account for uncertainty, biological variability (e.g., variable intraspecific divergence rates) and potential over‐splitting or over‐conservative species boundaries due to recent radiations (see Maroni et al. 2022; following Ranasinghe et al. 2022; Solovyeva et al. 2023).

Additional species delimitation was executed using the Poisson tree processes (PTP) method that determines species based on the number of substitutions between nodes (Zhang et al. 2013). The COI Bayesian and maximum likelihood (IQ‐TREE) phylogenetic trees obtained above were used as the input files for the bPTP web interface (https://species.h‐its.org/ last accessed October 2024). Two PTP methods were used (Zhang et al. 2013): first, the maximum likelihood (mlPTP) and second, the Bayesian (bPTP). The outgroup was removed, and the bPTP ran for 500,000 MCMC generations, with a burn‐in of 25% and a thinning value of 100 (as in Solovyeva et al. 2023). The trace plots were analyzed for both the bPTP and mlPTP output (Ranasinghe et al. 2022).

#### 2.3.2 Divergence Time Estimation

A Fossilized Birth Death model integrates fossil records (including extinct lineages) into the analysis by modelling speciation, extinction, and fossilization processes together, leading to more accurate and less biased divergence time estimates than node calibration priors (Heath et al. 2014). Therefore, an uncorrelated relaxed lognormal clock, random starting trees and Fossilized Birth Death model were used to estimate the divergence times of the two *Labidiaster* species for the COI dataset (following Heath et al. 2014; Jowers et al. 2023). These were implemented in BEAST2 version 2.7.6 (Bouckaert et al. 2019) using HKY + F + G4 as the best‐fit model. BEAUTi2 version 2.7.6 (Bouckaert et al. 2019) was used to generate XML input files for BEAST2 to calculate the Fossilized Birth Death model. Twenty million MCMC generations were sampled every 1000 generations. Trace plots were used to ensure convergence by examining the ESS (> 200). TreeAnnotator (Drummond and Rambaut 2007) was used to summarize the 95% highest posterior densities (HPD) and the maximum‐clade‐credibility tree topology with median ages. FigTree was used to view the tree, posterior probability and divergence times. The divergence times were calibrated with fossil evidence for Forcipulatacea (Asteroidea: Echinodermata) (Blake et al. 2017; Mah and Foltz 2011). Trichasteropsida represents an early‐diverging lineage within Forcipulatacea, with the fossil evidence dating back to 252.16 million years ago (mya) (Blake and Hagdorn 2003; Mah and Foltz 2011; Villier et al. 2018). Within the order Forcipulatida, a *Heliaster microbrachius* Xantus, 1860 fossil from the Pliocene in Florida, USA, 3–2.2 mya (Jones and Portell 1988) was used to calibrate the *Heliaster* node.

#### 2.3.3 Genetic Diversity and Structure

Phylogenetic and species delimitation analyses identified two distinct species‐level clades within *Labidiaster*. To avoid confounding effects in population‐level analyses, we analyzed these two putative species separately in all subsequent analyses.

To determine population structure and relationships among haplotypes within species, DnaSp was used to generate haplotype data files and diversity statistics. The number of haplotypes, haplotypic and nucleotide diversity, average number of nucleotide differences, number of polymorphic sites, and number of private haplotypes were generated in DnaSp. The diversity statistics for the COI and phased intron 7 datasets were obtained for both *Labidiaster* species total and within each geographic location.

The haplotype data output files from DnaSp for the COI and phased intron 7 datasets were used to generate TCS haplotype networks (Clement et al. 2000) at a 95% cutoff criterion using PopART version 1.7 (Leigh and Bryant 2015).

Arlequin version 3.5.2.2 (Excoffier and Lischer 2010) was used to calculate the genetic differentiation among locations (*F*_ST_). The *p*‐values were adjusted using the Bonferroni correction for multiple locations (Rice 1989). The R version 4.4.1 (R Core Team 2024) package “pegas” version 1.3 (Paradis et al. 2009) was used to carry out the Analysis of Molecular Variance (AMOVA) to determine the level of genetic differentiation among and within locations, for both the COI and phased intron 7 datasets and separately for *Labidiaster* species. Locations within the COI dataset with fewer than three individuals were excluded from the AMOVA (as in Maroni and Wilson 2022). For intron 7 datasets, locations with less than six phased sequences were removed from the AMOVA analyses. The genetic differentiation (*F*_ST_) among locations for all datasets was visualized in a heatmap using the R package “ggplot2” version 3.3.5 (Wickham 2016).

#### 2.3.4 Population Demographic History

Tajima's D and Fu's Fs statistic neutrality tests were both calculated using Arlequin. To estimate past population sizes over time, Bayesian Skyline Plots were implemented in BEAST2 (Bouckaert et al. 2019). The best‐fit models for all Bayesian Skyline Plots were obtained from the IQ‐TREE web server (http://iqtree.cibiv.univie.ac.at/ last accessed on 10 June 2024) using ModelFinder (Kalyaanamoorthy et al. 2017). The Bayesian information criterion (BIC) identified the best‐fit model for the COI *Labidiaster* species (clade I and II), as TN + F + I and HKY + F + I, respectively. The best‐fit model for the intron 7 dataset were identified as HKY + F and HKY + F + I for *Labidiaster* species (clade I and II), respectively. BEAUTi2, was used to generate XML input files for BEAST2 to calculate the Bayesian Skyline Plot (Bouckaert et al. 2019). A strict clock model was selected (Keogh et al. 2021; Miller et al. 2013), which assumes population size changes over time are of a constant evolutionary rate and a clock model rate (substitution rate) of 2.8% per site, per million years, was employed for COI following the rate for the class Asteroidea (Pérez‐Portela et al. 2017) and the phylum Echinodermata (Loeza‐Quintana et al. 2019). As the intron 7 genes are slower evolving at 29% of the rate of COI (Foltz, Bolton, et al. 2007), the clock model rate (substitution rate) of 0.812% (i.e., 0.028*0.29 = 0.00812) per million years was employed for intron 7. A coalescent Bayesian Skyline population tree prior was selected for all plots; 100 million (*Labidiaster* species clade I) and 200 million (*Labidiaster* species clade II) Markov chain Monte Carlo (MCMC) were run and sampled every 1000 generations. Additionally, a coalescent extended Bayesian Skyline population tree prior was also selected as a multi‐locus approach (combined COI and intron 7), using the same models, rates and parameters as above. Tracer was used to confirm convergence (ESS > 200), and both Tracer and R were used to visualize the Bayesian Skyline Plots.

#### 2.3.5 Isolation‐By‐Distance and Isolation‐By‐Depth

To determine whether genetic diversity is correlated with geographical distances, the genetic differentiation among locations (*F*_ST_), calculated in Arlequin, was plotted against the geographic distance among locations, followed by a partial Mantel test to determine whether genetic distances among individual sequences are correlated with geographical distances or depth among individual sequences.

To obtain the geographical distances (km), the least‐cost oceanic path (avoiding the Antarctic continent) was calculated. Southern Ocean bathymetry data (obtained from Kelly 2019) were plotted using “marmap” version 1.0 (Pante et al. 2013) and “raster” version 3.6–26 (Hijmans 2010) packages in R. The latitude and longitude coordinates from individual sequences (and locations) were converted to polar projections (Antarctic Polar Stereographic EPSG:3031) using the R packages “dplyr” version 1.1.4 (Wickham et al. 2023) and “sf” version 1.0–16 (Pebesma 2018; Pebesma and Bivand 2023). Using the “marmap” package in R, the least‐cost‐path distance (km) among individuals (and locations) was calculated and plotted to visually ensure that all paths are oceanic and do not overlap on land, and then the distances between locations and sequences (km) were converted to matrices. The genetic differentiation (*F*_ST_) and linearized *F*_ST_ (*F*_ST_/(1‐*F*_ST_)) among locations were then plotted against distances between locations using the “ggplot” package in R for both *Labidiaster* species, COI and intron 7. The linear regression line, standard errors, and the linear regression line equation, the coefficient of determination (*R* ^2^) and its associated significance (*p*‐value) were calculated for each plot.

Southern Ocean benthic invertebrates have been found to exhibit isolation by distance (e.g., Baird et al. 2012), and depth (e.g., *Pareledone* spp. in Lau et al. 2024; Strugnell et al. 2017). Eurybathy (the ability to survive in wide depth ranges) is suggested to be common among Southern Ocean benthic invertebrates (Brey et al. 1996), and this could also be the case for *Labidaster* species, as samples were collected from a large depth range. To visualize the depth ranges for each *Labidaster* species, the “ggplot” R package was used to construct a box and whisker plot from the individual sampling depths.

To determine whether there is a relationship between genetic distance and geographical distance (km) while controlling for the effect of depth (m), a partial Mantel test statistic and statistical significance for COI and intron 7 were calculated for both *Labidiaster* species. Additionally, a partial Mantel test was used to explore the relationship between genetic distance and depth while controlling for geographical distance. The pairwise genetic distances among individual sequences (K80 model) were determined using “ape” version 5.8 (Paradis et al. 2002) package in R. These genetic distances were used together with the least‐cost‐path distance (km) matrix and depths (m) among individuals to conduct partial Mantel tests for Pearson, Spearman, and Kendall models in R using “vegan” version 2.6–6.1 (Oksanen et al. 2001), using 9999 permutations.

#### 2.3.6 Gene Flow

The mtDNA (COI) datasets were used to infer historical dispersal between locations within species using migrate‐n version 5.0.4 (Beerli et al. 2022, 2019). Migrate‐n estimates gene flow based on population size (θ = 4Neμ, where *μ* is the mutation rate per generation and Ne is the effective population size) and the immigration rate (*M* = *m*/*μ*, where m is the immigration rate per generation) (Beerli and Palczewski 2010). The same best‐fit models used for the COI Bayesian Skyline Plots for each *Labidiaster* species were also used for the migrate‐n analyses.

**FIGURE 1:**
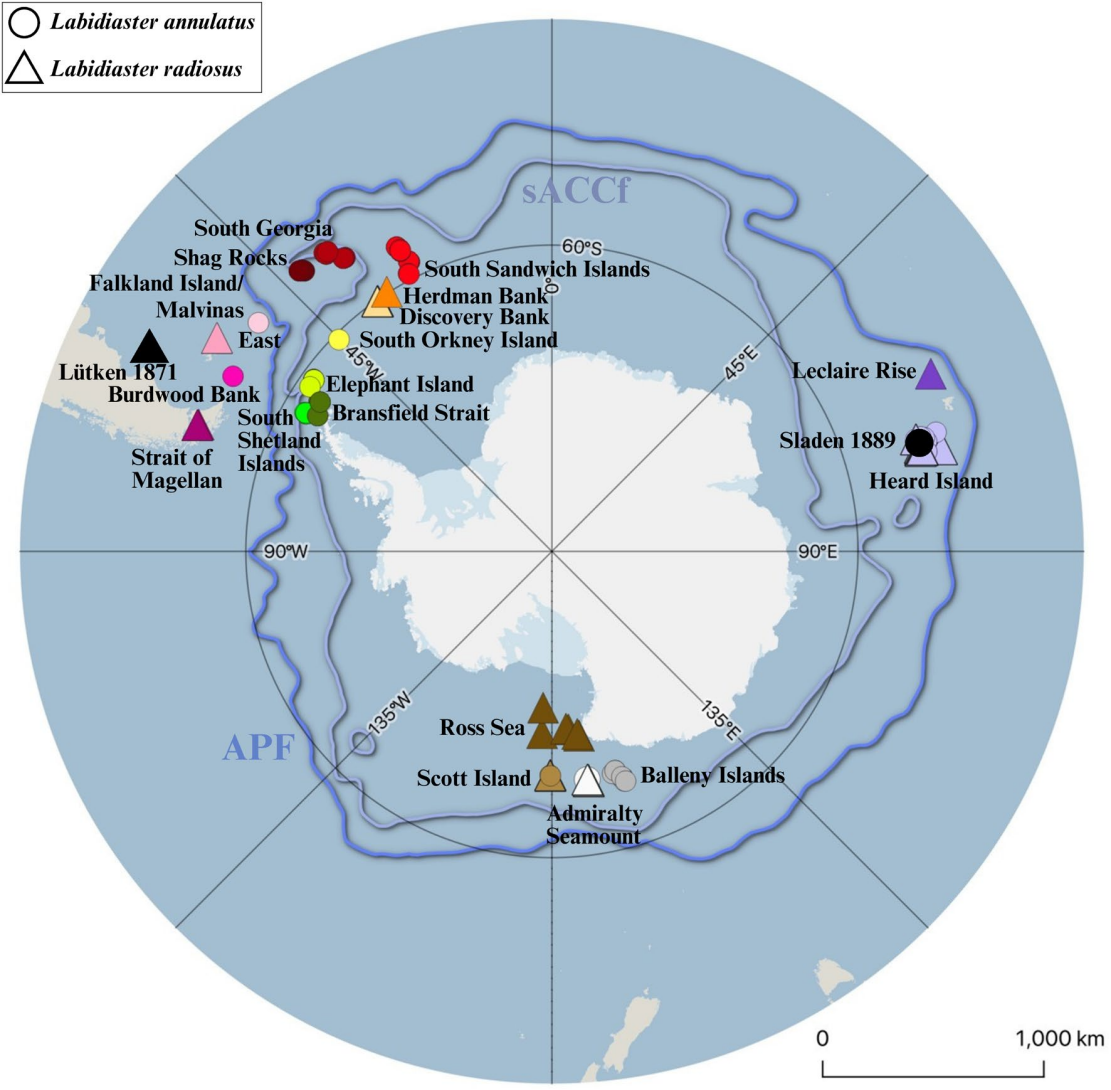
Sample locations of *Labidiaster annulatus* (circle) and *Labidiaster radiosus* (triangle) sequences in the Southern Ocean. Colors correspond to different sample locations. The type locality of *Labidiaster annulatus* (black circle) and *Labidiaster radiosus* (black triangle) is indicated. Blue lines indicate frontal system borders AFP, Antarctic Polar Front; sACCf, Southern Antarctic Circumpolar Current Front (Quantarctica: Matsuoka et al. 2021). For corresponding sample metadata see Table A2.

The migrate‐n analysis focused on *L. annulatus* Scotia Arc and Antarctic Peninsula populations. These were grouped into 10 main populations according to geographical locations: (1) Shag Rocks, (2) South Georgia West, (3) South Georgia East, (4) Visokoi Island (South Sandwich Island), (5) Candlemas Island (South Sandwich Island), (6) Montagu Island (South Sandwich Island), (7) Elephant Island, (8) South Shetland Islands, (9) Bransfield Strait Mouth, and (10) Bransfield Strait. *Labidaster radiosus* populations were grouped into five main populations according to geographical locations: (1) Strait of Magellan, (2) Falkland Islands/Malvinas, (3) Discovery and Herdman Banks, (4) Heard Island, and (6) Ross Sea Region (Scott Island, Ross Sea and Admiralty Seamount) (Figure 1).

The migrate‐n analysis for each *Labidiaster* species was run using one long chain with 10 replicates, 1 million recorded steps with 1000 sampling increments and 10% as burn‐in (Čekovská et al. 2020; Cortez et al. 2020; Jordan et al. 2024; Li et al. 2024). A four‐chain static heating scheme was selected with temperatures of 1.00, 1.50, 3.00, and 10 million (Crandall et al. 2019; Jordan et al. 2024; Li et al. 2024). Tracer was used to confirm convergence and that ESS numbers were greater than 200 (Beerli et al. 2022, 2019). The Bezier log (mL) was used from each model to calculate the log Bayes factors (LBF) and the likelihood of each model occurring (probability). The number of immigrants per generation (N*m*); N*m* = (θ_receiving population_ * *M*_j → i_)/*x* were calculated for haploid (such as mtDNA) data with *x* = 2 (Beerli et al. 2019; Beerli and Palczewski 2010; Fourdrilis and Backeljau 2019). For *L. annulatus*, the uniform Bayesian priors were implemented (θ: 0 0.7 0.07; *M*: 0 15000 1500). For *L. radiosus*, the uniform Bayesian priors were implemented (θ: 0 0.8 0.08; *M*: 0 15000 1500).

The following migrate models were implemented in migrate‐n to infer gene flow between populations (e.g., Bucklin et al. 2021; Čekovská et al. 2020; Crandall et al. 2019; Fourdrilis and Backeljau 2019): For *L. annulatus* ; Model (1) m migration, (2) panmixia, (3) north to south stepping‐stone and (4) south to north stepping‐stone. For *L. radiosus* ; Model (1) m migration, (2) panmixia, (3) unidirectional clockwise, (4) unidirectional anticlockwise, and (5) stepping‐stone. Within each *Labidiaster* species, all migration models were run with the same parameters and priors.

Using the same COI datasets and location groupings as for the migrate‐n analyses, Genepop version 4.7.5 (Raymond and Rousset 1995; Rousset 2008) was used to estimate the effective number of migrants (N*m*) corrected for sample size (Barton and Slatkin 1986) among locations, inferring historical dispersal. However, the directionality of migrants is not indicated.

To infer contemporary dispersal between locations, BayesAss version 3.0.5.6 (Rannala 2007) was used to estimate the recent migration rates for the COI datasets. Five replicates of 10 million runs were included with 10% burn‐in, sampling every 1000 iterations (Gomes et al. 2024; Vargas‐Fonseca et al. 2021).

## 3 Results

A total of 364 COI sequences (29 haplotypes) and 650 phased (16 haplotypes) intron 7 sequences were included in the final alignment length, which comprised 704 base pairs (bp) and 388 bp, respectively (Table A2).

### 3.1 Phylogenetic Reconstruction

**FIGURE 2:**
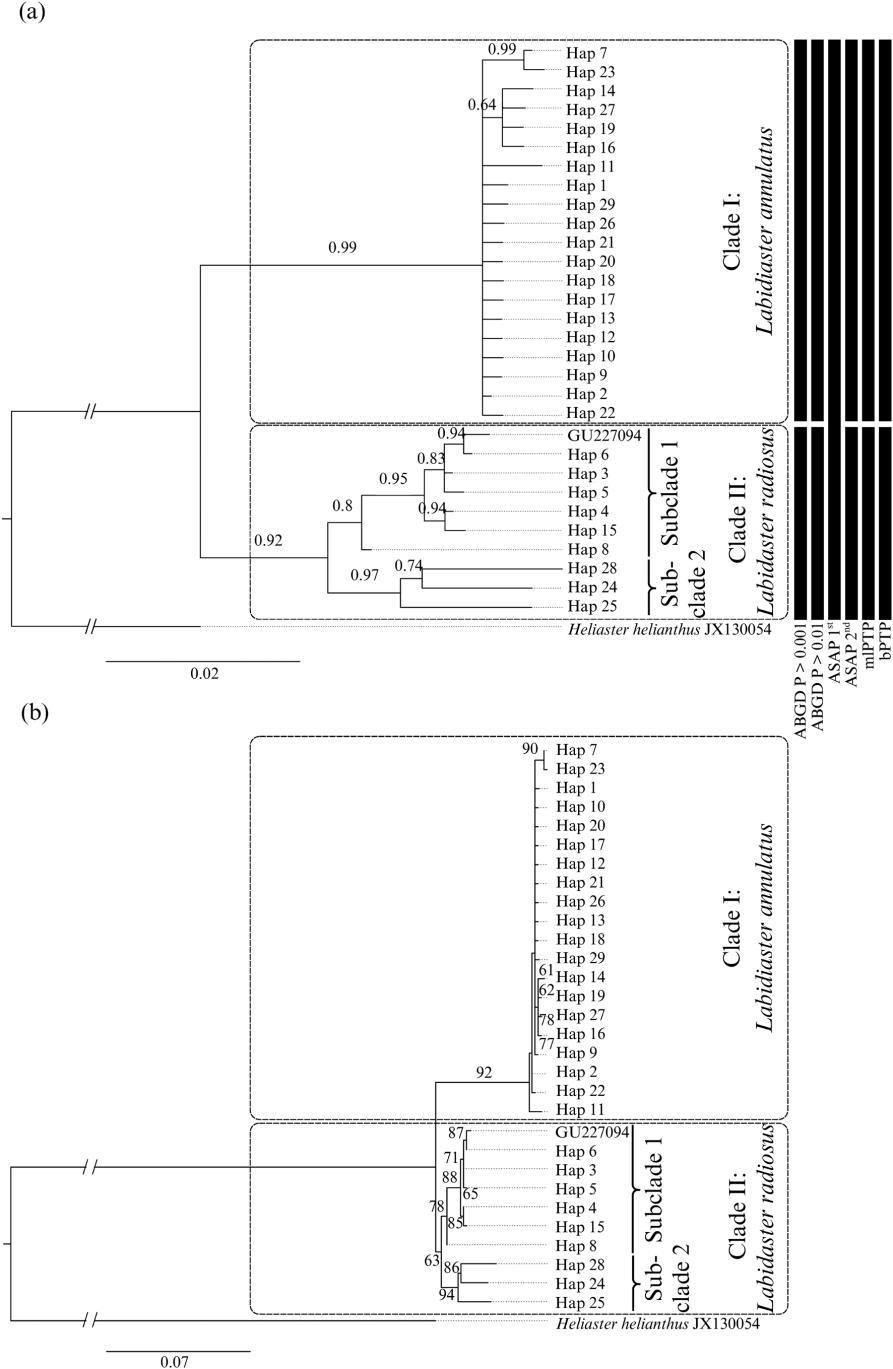
Phylogenetic trees showing haplotypes for *Labidiaster* spp. from COI sequences with *Heliaster helianthus* serving as the outgroup taxa. (a) Bayesian posterior probabilities and (b) maximum likelihood bootstrap support ≥ 50% given on the branch. Species delimitation analyses are indicated by black bars on the right side of the Bayesian phylogenetic tree (a). Dashed boxes indicate *Labidiaster* clades and open brackets show subclades within *Labidiaster* Clade II. The scale bar represents branch lengths based on nucleotide substitutions per site.

The mtDNA (COI) Bayesian phylogenetic tree (Figure 2a) topology reveals two monophyletic clades corresponding to the morphological species *L. annulatus* (clade I) and *L. radiosus* (clade II), supported by high and moderate posterior probabilities of 0.99 and 0.82, respectively. The maximum likelihood (Figure 2b) topology revealed one well‐supported monophyletic clade for species *L. annulatus* (clade I, 92%) and a second, less well‐supported clade *L. radiosus* (clade II, 63%). Within *L. radiosus* (clade II), the COI tree topologies show two subclades; the first (subclade 1) included samples located south of the APF and the unidentified *Asteriidae* sp. bipinnaria larvae sequence (GenBank: GU227094), supported by moderate bootstrap and posterior probability of 78% and 0.8 (Figure 2a,b). The second subclade (subclade 2) included samples located north of the APF (southern South America) and was likewise supported by high bootstrap and posterior probability of 94% and 0.97, respectively (Figure 2a,b). The sister‐species (clade I and II) were also supported by species delimitation (Figure 2a) analyses, including ABGD at 0.001 < *p* > 0.01 (upper and lower limit), ASAP 2nd, lPTP, and bPTP. ASAP 1st did not reveal intraspecific distance as it grouped all *Labidiaster* sequences as a single species.

**FIGURE 3:**
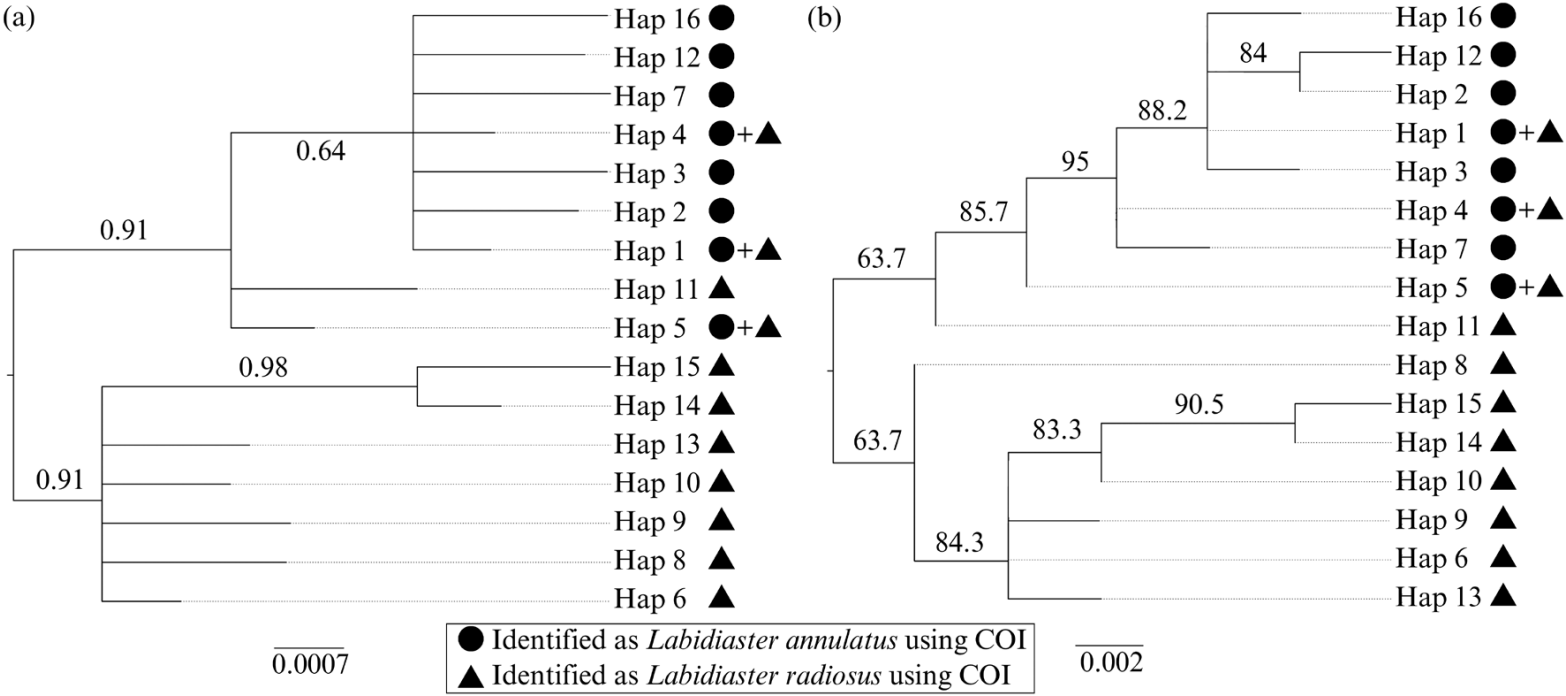
Phylogenetic trees showing haplotypes for *Labidiaster* spp. from intron 7 sequences with midpoint rooting. (a) Bayesian posterior probabilities and (b) maximum likelihood bootstrap support given on the branch. The scale bar represents branch lengths based on nucleotide substitutions per site.

The nuDNA (intron 7) Bayesian phylogenetic trees (Figure 3a) and maximum likelihood (Figure 3b) topology do not show the same groupings, nor the separation of the two *Labidiaster* species. Both Bayesian and maximum likelihood intron 7 tree topologies reveal two monophyletic clades supported by moderate bootstrap and high posterior probabilities of 62% and 0.91, respectively. The intron 7 trees did not show the same separation as the COI trees for *L. annulatus* (clade I) and *L. radiosus* (clade II), nor for the subclades in *L. radiosus* (clade II) (i.e., south and north of the APF). However, three haplotypes (Hap_1, Hap_4 and Hap_5) were shared between *Labidiaster* species identified with COI (Figure 3a,b).

Divergence time (Figure S2) between *L. annulatus* (clade I) and *L. radiosus* (clade II) based on COI data was estimated at ~3.56 mya (95% highest posterior density = 5.25–2.15). The *Heliaster* outgroup was estimated to have diverged ~4.58 mya (HPD95: 6.64–3.13) from the *Labidiaster* genus. *Labidiaster radiosus* (clade II) south of the APF diverged from those north of the APF ~2.08 mya (HPD95: 3.48–0.58).

### 3.2 Genetic Structure

**FIGURE 4:**
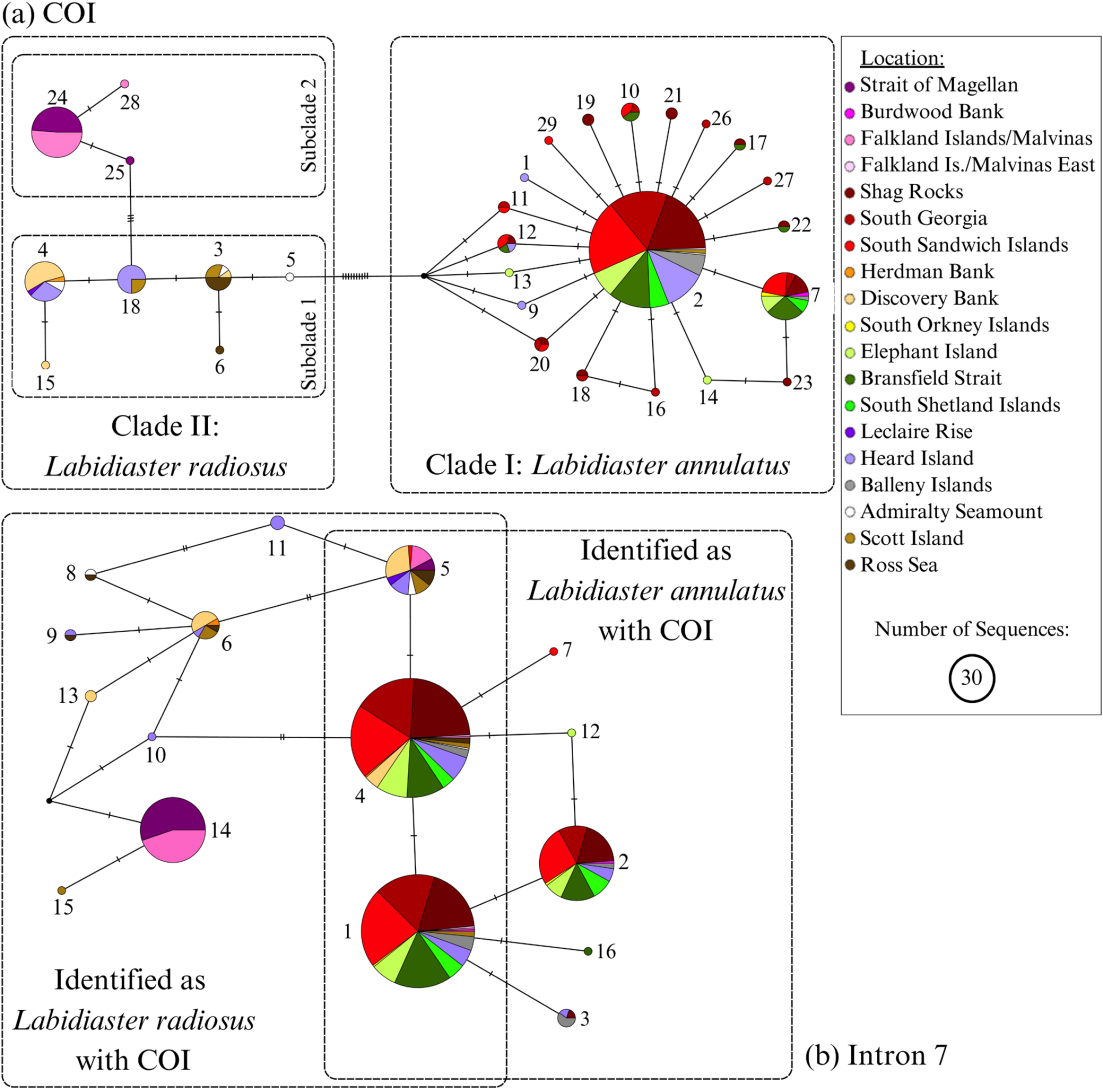
TCS haplotype network of *Labidiaster annulatus* (Clade I, dashed box) and *Labidiaster radiosus* (Clade II, dashed box). (a) COI sequences, and within *Labidiaster radiosus*, Subclade 1 includes sequences south of the Antarctic Polar Front and Subclade 2 includes sequences north of the Antarctic Polar Front (shown in boxes). (b) Intron 7 sequences and shared haplotypes between species identified with COI are shown in overlapping boxes. Each location is represented by a different color corresponding to Figure 1. Circle sizes are proportional to the number of sequences in each haplotype. Lines perpendicular to the connecting lines indicate substitutions, and black dots indicate missing haplotypes.

The COI dataset comprised 29 haplotypes, including 13 private haplotypes (Figure 4a). The TCS network for COI (Figure 4a) shows that *L. annulatus* and *L. radiosus* are separated by 10 substitutions, reflecting the species delimitation and phylogenetic clades for the COI dataset. The TCS network revealed *L. annulatus* haplotypes forming a “star‐like” network (see Allcock and Strugnell 2012), with a frequent (*n* = 206) and one less frequently recovered (*n* = 34) common haplotype. Specimens collected south and north of the APF, including those from Burdwood Bank (*n* = 1) and east of the Falkland Islands/Malvinas, share the same dominant haplotypes. For *L. radiosus* , the network showed some structuring by location; shared haplotypes north of the APF (Falkland Islands/Malvinas and Strait of Magellan) were separated by three substitutions from *L. radiosus* south of the APF.

The intron 7 dataset comprised 16 haplotypes with 7 private haplotypes (Figure 4b). The TCS network (Figure 4b) revealed some shared haplotypes between COI identified *L. annulatus* or *L. radiosus* (Haplotypes 1, 4 and 5).

### 3.3 Genetic Diversity

The overall average COI haplotypic diversity (0.7184 ± 0.031) and nucleotide diversity (0.0115 ± 0.0003) for *L. radiosus* (Table 1) were higher than for *L. annulatus* (0.4197 ± 0.0360, 0.0022 ± 0.0002, Table 2). However, more private haplotypes (*n* = 9) were detected in *L. annulatus* than in *L. radiosus* (*n* = 5). Within the *L. annulatus* COI dataset (Table 2), Shag Rocks (*n* = 54), Elephant Island (*n* = 21) and Bransfield Strait (*n* = 38) had the highest haplotypic (0.7000 ± 0.0670, 0.8950 ± 0.0470, 0.7480 ± 0.0580) and nucleotide diversity (0.00263 ± 0.00038, 0.00391 ± 0.0006, 0.00227 ± 0.00035). Within the *L. radiosus* COI dataset (Table 1), the Strait of Magellan (*n* = 20) had the lowest haplotypic and nucleotide diversity (0.1000 ± 0.0880, 0.00024 ± 0.0002). Admiralty Seamount (*n* = 4) had the highest haplotypic and nucleotide diversity (1.0000 ± 0.1770, 0.0040 ± 0.0009).

**TABLE 1:** Genetic diversity indices and neutrality tests for *Labidiaster radiosus* . Locations for COI with *n* < 3 were removed (Herdman Bank, Leclaire Rise), and for intron 7 with *n* < 6 were removed (Herdman Bank, Leclaire Rise, Admiralty Seamount). Tajima's *D* and Fu's Fs are statistically significant at *p* < 0.05, indicated by an asterisk (*). The sample size (*n*), haplotypic diversity (*H*), nucleotide diversity (π), number of polymorphic sites (*S*), and standard deviations (SD) are shown in the columns.

Location	*n*	Number of haplotypes	*H*	*H* ± SD	π	π ± SD	*S*	Number of private haplotypes	Tajima's *D*	Tajima's *D p*	Fu's Fs	Fu's Fs *p*
*COI*												
Strait of Magellan	20	2	0.1000	0.0880	0.0002	0.0002	1	1	−1.164	0.115	−0.879	0.101
Falkland Islands/Malvinas	21	3	0.2670	0.1200	0.0007	0.0003	2	1	−1.159	0.146	−1.259	0.033*
Discovery Bank	15	3	0.2570	0.1420	0.0012	0.0007	3	1	−1.685	0.021*	−0.831	0.079
Heard Island	16	2	0.5250	0.0550	0.0011	0.0011	1	0	1.474	0.900	1.333	0.755
Admiralty Seamount	4	4	1.0000	0.1770	0.0040	0.0009	5	1	0.372	0.637	−1.322	0.136
Scott Island	6	2	0.6000	0.1290	0.0021	0.0005	2	0	1.753	0.924	1.938	0.868
Ross Sea	6	3	0.6000	0.2150	0.0010	0.0004	2	1	−1.295	0.064*	0.297	0.512
Total	90	9	0.7184	0.0310	0.0115	0.0003	12	5	−0.104	0.595	−0.055	0.350
*Intron 7*												
Strait of Magellan	40	2	0.1420	0.0710	0.0018	0.0009	5	0	−1.004	0.170	2.647	0.876
Falkland Islands/Malvinas	38	4	0.3600	0.0890	0.0044	0.0010	6	1	0.498	0.663	2.199	0.922
Discovery Bank	28	4	0.5050	0.0940	0.0037	0.0007	4	1	1.040	0.831	1.291	0.809
Heard Island	12	6	0.8030	0.0960	0.0047	0.0011	5	0	0.328	0.621	−1.607	0.089
Admiralty Seamount	4	3	0.8330	0.2220	0.0052	0.0020	4	0	−0.780	0.174	0.134	0.329
Scott Island	10	5	0.7560	0.1300	0.0055	0.0013	6	1	0.024	0.509	−0.455	0.381
Ross Sea	10	4	0.6440	0.1520	0.0046	0.0012	5	0	0.074	0.510	0.361	0.599
Total	146	10	0.6650	0.0260	0.0066	0.0002	8	3	0.020	0.609	0.630	0.600

**TABLE 2:** Genetic diversity indices and neutrality tests for *Labidiaster annulatus* . Locations for COI with *n* < 3 were removed (Burdwood Bank, Falkland Islands/Malvinas East, South Orkney Islands, Admiralty Seamount, Scott Island), and for intron 7 with *n* < 6 were removed (Burdwood Bank, Falkland Islands/Malvinas East, South Orkney Islands, Scott Island). Tajima's D and Fu's Fs are statistically significant at *p* < 0.05, indicated by an asterisk (*). The sample size (*n*), haplotypic diversity (*H*), nucleotide diversity (π), number of polymorphic sites (*S*), and standard deviations (SD) are shown in the columns.

Location	*n*	Number of haplotypes	*H*	*H* ± SD	π	π ± SD	*S*	Number of private haplotypes	Tajima's *D*	Tajima's *D p*	Fu's Fs	Fu's Fs *p*
*COI*												
Shag Rocks	54	15	0.7000	0.0670	0.0026	0.0004	14	3	−2.066	0.003*	−13.837	0.000*
South Georgia	43	9	0.3780	0.0950	0.0013	0.0004	7	2	−2.027	0.000*	−9.607	0.000*
South Sandwich Islands	58	7	0.4360	0.0760	0.0017	0.0003	6	0	−1.555	0.032*	−4.638	0.002*
Elephant Island	21	11	0.8950	0.0470	0.0039	0.0006	12	2	−1.510	0.034*	−6.075	0.000*
South Shetland Islands	14	3	0.6260	0.1040	0.0014	0.0003	2	0	0.416	0.634	0.206	0.551
Bransfield Strait	38	10	0.7480	0.0580	0.0023	0.0004	10	0	−1.527	0.032*	−5.219	0.001*
Heard Island	27	6	0.4530	0.1116	0.0012	0.0004	5	2	−1.573	0.012*	−3.628	0.000*
Balleny Islands	13	3	0.2950	0.1560	0.0010	0.0005	2	0	−1.468	0.043*	−1.402	0.019*
Total	268	20	0.4197	0.0360	0.0022	0.0002	17	9	−3.078	0.002*	−2.342	0.05*
*Intron 7*												
Shag Rocks	108	4	0.6250	0.0240	0.0020	0.0001	3	0	0.680	0.771	0.824	0.698
South Georgia	84	3	0.6120	0.0240	0.0019	0.0001	2	0	1.321	0.879	1.608	0.810
South Sandwich Islands	116	5	0.6580	0.0180	0.0022	0.0001	4	1	0.233	0.631	0.092	0.554
Elephant Island	42	4	0.6550	0.0380	0.0021	0.0002	2	1	1.452	0.919	0.234	0.520
South Shetland Islands	26	3	0.6890	0.0300	0.0023	0.0002	2	0	1.480	0.932	1.185	0.730
Bransfield Strait	70	4	0.6440	0.0290	0.0020	0.0002	3	1	0.495	0.733	0.491	0.597
Heard Island	30	5	0.6940	0.0490	0.0024	0.0003	4	0	−0.188	0.431	−0.805	0.276
Balleny Islands	18	4	0.7250	0.0690	0.0024	0.0004	3	0	0.178	0.624	−0.293	0.383
Total	504	8	0.6460	0.0090	0.0021	0.0000	6	3	0.420	0.774	0.292	0.571

For the intron 7 datasets, similar overall haplotypic diversity (Tables 1 and 2) was found for *L. annulatus* and *L. radiosus* (0.6460 ± 0.0090, 0.6650 ± 0.0260), with three private haplotypes. The total intron 7 nucleotide diversity for *L. radiosus* (0.0066 ± 0.0002) was slightly higher than for *L. annulatus* (0.0021 ± 0.0000). Within the *L. annulatus* , Balleny Islands (*n* = 18) had the highest haplotypic diversity (0.7250 ± 0.0690). The remaining locations had comparable haplotypic diversities to each other. Within the *L. radiosus* , Heard Island (*n* = 12) had the highest haplotypic and nucleotide diversity (0.8030 ± 0.0960, 0.0047 ± 0.0011).

### 3.4 Genetic Differentiation

For *L. radiosus* , the AMOVA revealed significant (*p* = 0.00) genetic differentiation (Table S1) between locations in both COI and intron 7 datasets (*F*_ST_ = 0.9939 and *F*_ST_ = 0.5108, respectively). For COI, the differentiation among locations was 92.4%, which was higher than the differentiation within locations, at 7.6%. This differed from the slower evolving intron 7, for which the differentiation among locations was 51.1%; this was similar to the differentiation within locations, at 48.9%.

**FIGURE 5:**
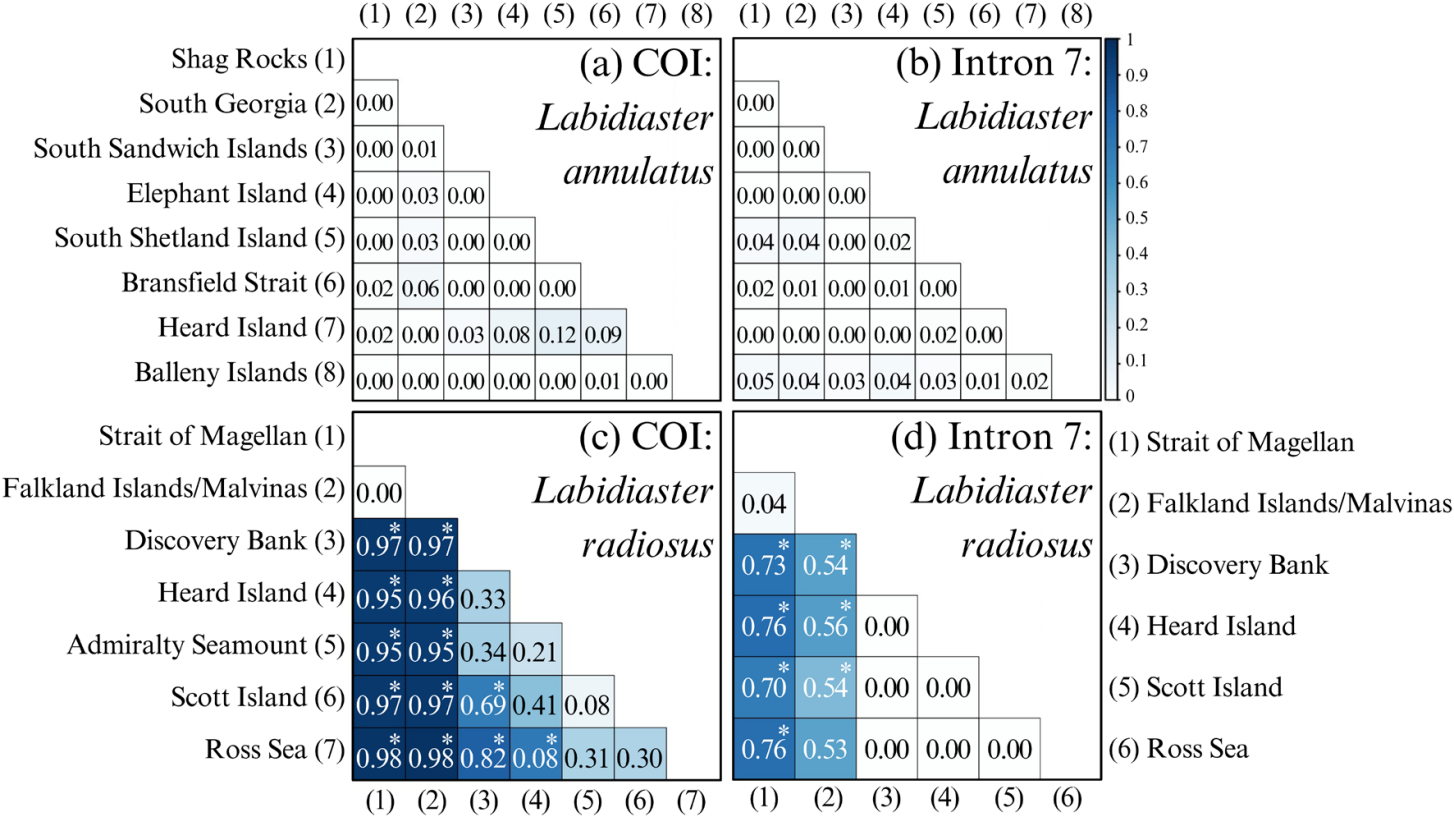
Heatmap visualizing pairwise *F*_ST_ values among locations for *Labidiaster annulatus* (a) COI and (b) Intron 7 dataset. Locations for COI with *n* < 3 (Burdwood Bank, Falkland Islands/Malvinas East, South Orkney Islands, Admiralty Seamount, Scott Island) and for intron 7 with *n* < 6 (Burdwood Bank, Falkland Islands/Malvinas East, South Orkney Islands, Scott Island) were not used in the plot. For *Labidiaster radiosus* (c) COI and (d) intron 7 dataset. Locations for COI with *n* < 3 (Herdman Bank, Leclaire Rise) and for intron 7 with *n* < 6 (Herdman Bank, Leclaire Rise, Admiralty Seamount) were not used in the plot. Bonferroni‐corrected significant *p*‐value (*p* < 0.002) is shown with an asterisk (*).

For *L. annulatus*, the AMOVA revealed nonsignificant (*p* > 0.1) genetic differentiation (Table S1) in both the COI and intron 7 datasets (*F*_ST_ = 0.0085 and *F*_ST_ = 0.0030, respectively). However, for COI, the differentiation among locations was 0.9%, which was lower than the differentiation within locations at 99.2%. This was similar to intron 7, which revealed that the differentiation among locations was 0.3%, which was also lower than the differentiation within locations at 99.7%. For *L. annulatus* , pairwise *F*_ST_ among locations (Figure 5a,b) revealed no significant (*p* > 0.002) differentiation for COI or intron 7.

Within *L. radiosus* , pairwise *F*_ST_ for COI (Figure 5c) revealed low and nonsignificant genetic differentiation between the Strait of Magellan and the Falkland Islands/Malvinas (*F*_ST_ = 0, *p* > 0.002). However, high and significant genetic differentiation was found among the Strait of Magellan and all other locations (*F*_ST_ > 0.95, *p* < 0.00) and among the Falkland Islands/Malvinas and all other locations (*F*_ST_ > 0.95, *p* < 0.00). Additionally, significant high genetic differentiation was found among locations Discovery Bank and Scott Island (*F*_ST_ = 0.69, *p* = 0.00), Ross Sea (*F*_ST_ = 0.82, *p* = 0.00), and also among Heard Island and Ross Sea (*F*_ST_ = 0.71, *p* = 0.00). Within *L. radiosus* , pairwise *F*_ST_ for intron 7 among locations (Figure 5d) revealed low and non‐significant genetic differentiation between Strait of Magellan and Falkland Islands/Malvinas (*F*_ST_ = 0.04, *p* > 0.002). However, very high and significant genetic differentiation was found among the Strait of Magellan and all other locations (*F*_ST_ > 0.70, *p* = 0.00) and among the Falkland Islands/Malvinas and all other locations (*F*_ST_ > 0.45, *p* = 0.00). For the intron 7 dataset, no significant genetic differentiation was revealed among any other locations.

### 3.5 Population Demographic History

**FIGURE 6:**
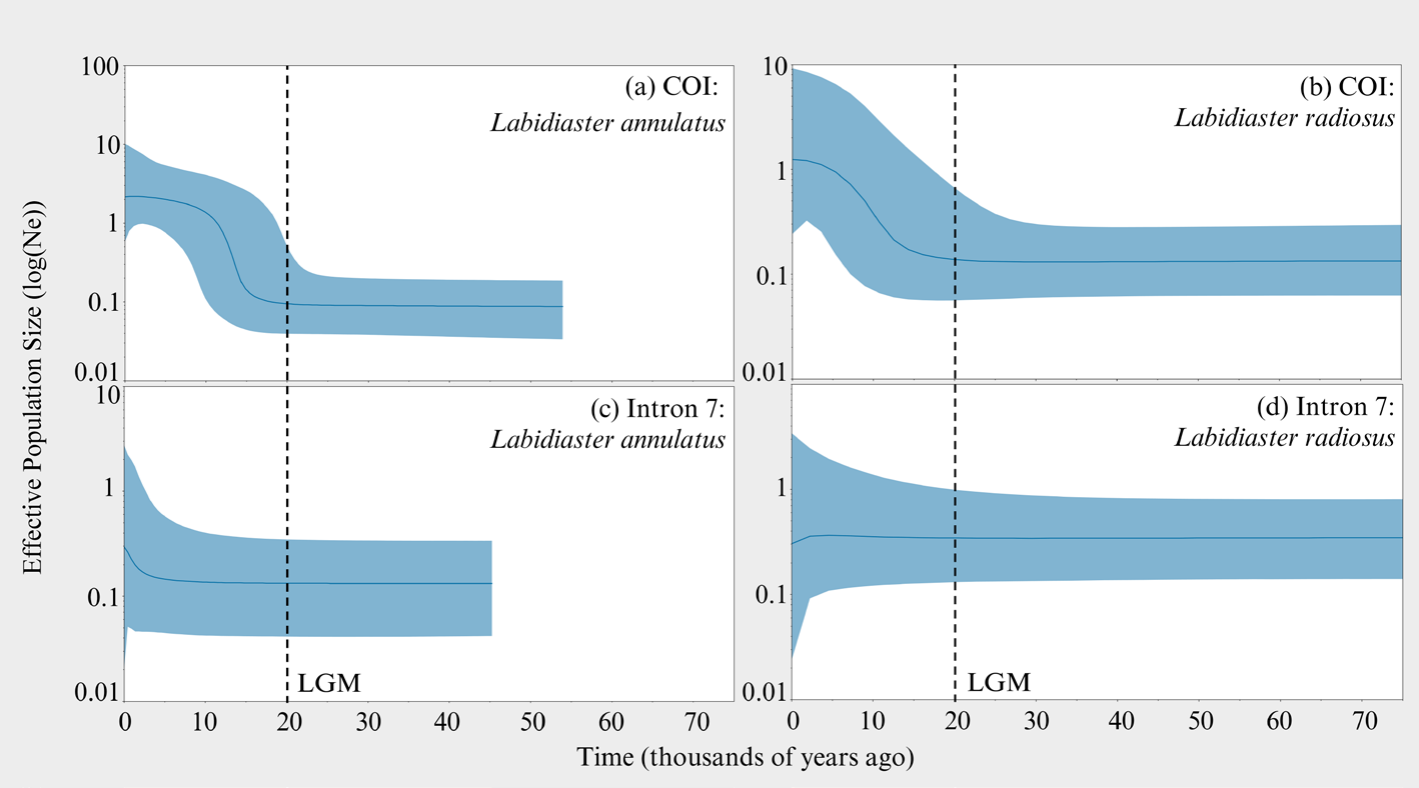
Coalescent Bayesian skyline plot showing effective population sizes (log(Ne)) across time (thousand years ago) for COI (a) *Labidiaster annulatus* , (b) *Labidiaster radiosus* and intron 7, (c) *Labidiaster annulatus,* (d) *Labidiaster radiosus* . The solid blue line represents the median, the blue shading on either side of the line represents 95% highest posterior density and the dashed vertical lines represent the time of the last glacial maximum (LGM, ~20,000 years ago McCave et al. 2014).

The Bayesian Skyline Plot for *L. annulatus* (COI) suggests a population expansion at 20,000 years ago, coinciding with the last glacial maximum (LGM timing in McCave et al. 2014) (Figure 6a). Furthermore, for all *L. annulatus* locations (except South Shetland Islands), population size changes were indicated by a significant negative Fu's Fs (Fu 1997), and past population expansions were indicated by a significant negative Tajima's *D* (Tajima 1989) (Table 2).

Evidence of possible population expansions for *L. radiosus* (COI) at Discovery Bank and Ross Sea locations was supported by significant negative Tajima's *D* values (−1.685, *p* = 0.021; −1.29503, *p* = 0.064*). However, these were nonsignificant for Fu's Fs (Table 1). The COI Bayesian Skyline Plot for *L. radiosus* (Figure 6b) is similar to the COI Bayesian Skyline Plot for *L. annulatus* (Figure 6a) and reveals a population expansion that occurred around the same time as the LGM.

Tajima's *D* and Fu's Fs values obtained for *L. annulatus* (intron 7) and *L. radiosus* (COI and intron 7) were not significant (Tables 1 and 2). The Bayesian Skyline Plot for *L. annulatus* (intron 7) indicates a slight population increase 5000 years ago (Figure 6c) and for *L. radiosus* a slight population reduction 5000 years ago (Figure 6d, combined BSP for *L. annulatus* and *L. radiosus* in Figures S3 and S4, respectively).

### 3.6 Isolation‐By‐Distance and Depth

Isolation‐by‐distance (IBD) was not detected within *L. annulatus* when comparing genetic distances between individual sequences or locations with geographic distances. The partial Mantel tests found no relationship between genetic distance and geographical distance while controlling for the effect of depth for both the COI and intron 7 datasets, across all locations and only focusing on the locations within Scotia Arc (Table A3). No relationship was also found between genetic differentiation (*F*_ST_) or linearized genetic distances and geographical distance among locations in the COI and intron 7 dataset for *L. annulatus* (Figure S5).

Isolation‐by‐distance (IBD) was detected within *L. radiosus* (COI and intron 7) when comparing genetic distances between individual sequences or locations with geographic distances. For both the COI and intron 7 datasets, partial Mantel tests revealed significant (*p* < 0.0001*) positive relationship between genetic distance and geographical distance, while controlling for the effect of depth (Table A3). Similarly, a significant (*p* < 0.0001*) positive relationship between genetic distance and depth was revealed, while controlling for the effect of geographical distance (Table A3). A significant positive relationship (*R* ^2^ = 0.3, *p* < 0.0108*) was also found for COI between genetic differentiation (*F*_ST_) and geographical distance among locations (Figure S6a). However, for COI no relationship was revealed between the linearized genetic differentiation (*F*_ST_/1‐*F*_ST_) and geographical distance among locations (Figure S6c), nor for intron 7 of *L. radiosus* (Figure S6b,d). Furthermore, for the *L. radiosus* subclade 1 (south of the APF only), the COI partial Mantel tests revealed a significant positive Pearson relationship between genetic distance and geographical distance but not for depth (Table A3).

### 3.7 Dispersal

**FIGURE 7:**
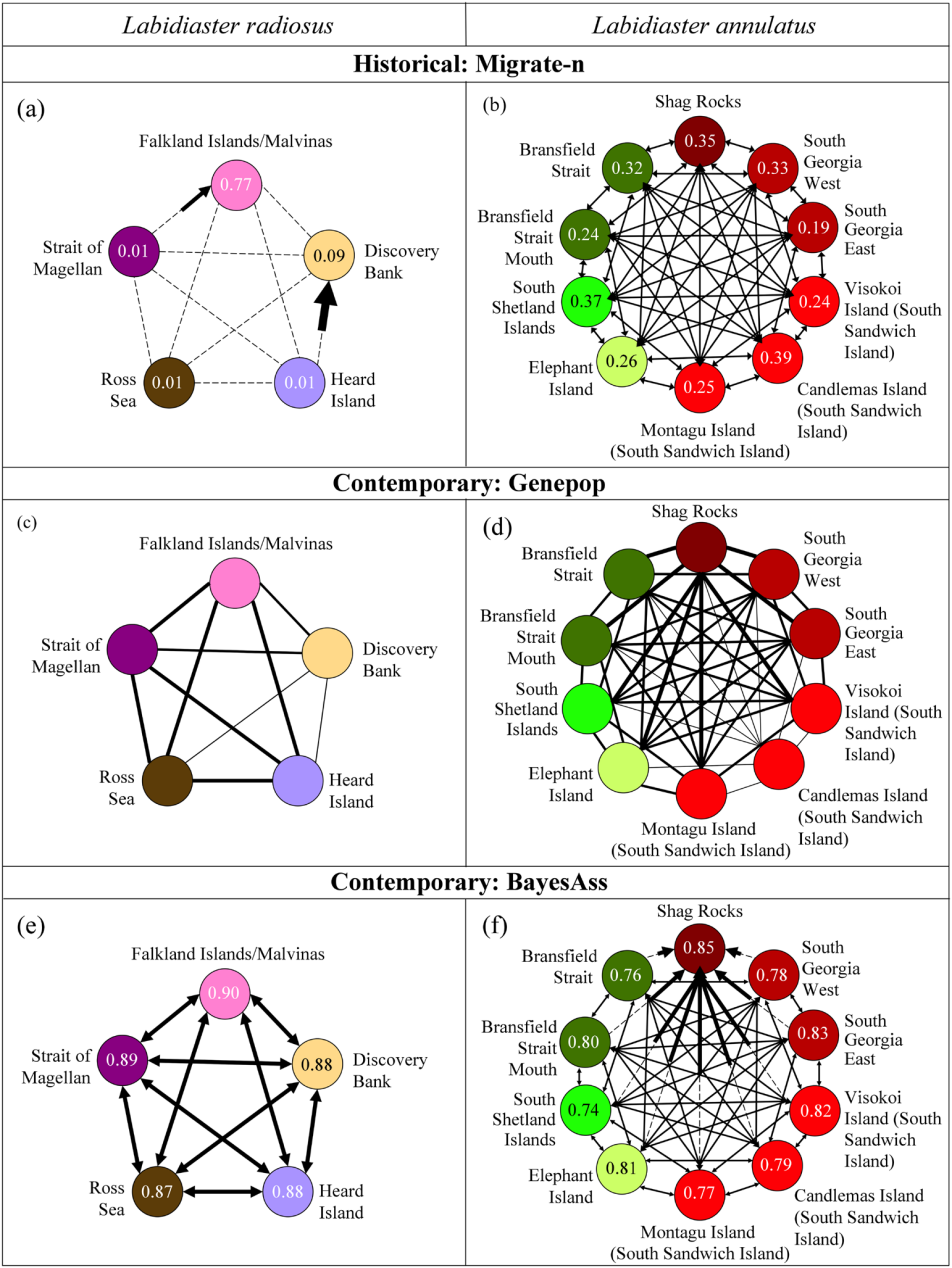
Migrate‐n results of the full migration model showing number of immigrants into receiving populations with 95% confidence intervals for (a) *Labidiaster radiosus* and (b) Scotia Arc locations of *Labidiaster annulatus* . Contemporary migration (Genepop) showing number of immigrants between populations for (c) *Labidiaster radiosus* and (d) Scotia Arc locations of *Labidiaster annulatus* . Contemporary migration (BayesAss) showing migration rate for (e) *Labidiaster radiosus* and (f) Scotia Arc locations of *Labidiaster annulatus*. Location colour corresponds to Figure 1 and arrows indicate migration direction. Significant migration is represented by a solid line (non‐significant by a dashed line), and the width of the line corresponds to the number of immigrants and migration rate. The retention rate is shown in the center of the circles.

For *L. annulatus* , migrate‐n analyses showed that the panmixia model had the highest probability in explaining historical dispersal (1.00, Table S2b). The historical migration analysis of the full migration model revealed significant migration rates with a high number of immigrants between all locations from the Antarctic Peninsula and across the Scotia Arc (Figure 7b). The high number of immigrants between locations (> 82.4, 95%: 54.5–109.1) showed that *L. annulatus* had historically high genetic connectivity and subsequent high dispersal.

The contemporary dispersal (genepop and BayesAss analyses) for *L. annulatus* among all locations revealed a moderate to high number of migrants (Figure 7d,f). Based on genepop, Candlemas Island (of the South Sandwich Islands group) showed the lowest number of migrants (N*m* < 1.47) and Shag Rocks the highest (N*m* > 3.46) among all locations (Figure 7d). On the basis of BayesAss, Shag Rocks received a large number of immigrants from all locations, except for moderate numbers from Candlemas Island (South Sandwich Island) and South Georgia East (Figure 7f).

For *L. radiosus* , the panmixia model (n‐migrate) for the historical dispersal received the highest probability (1.00, Table S3a). Historical dispersal revealed significant migration rates showing a high number of immigrants (Figure 7a) dispersing from the Strait of Magellan to the Falkland Islands/Malvinas (4050.7, 95%: 2308.7–4836.3). Additionally, significant migration rates showing a high number of immigrants dispersing from Heard Island to Discovery Bank, were shown (341.3, 95%: 203.7–422.9). The contemporary (genepop and BayesAss analyses) for *L. radiosus* revealed a lower number of migrants among locations than for *L. annulatus* (Figure 7c,e). Based on genepop, the number of immigrants was highest between the Strait of Magellan, the Falkland Islands/Malvinas and the Ross Sea region (Figure 7c).

## 4 Discussion

### 4.1 What Is the Geographic Distribution and Genetic Relationship of *L. radiosus* and *L. annulatus* ?

The geographic distributions of *L. annulatus* and *L. radiosus* presented in this study extend their previously documented distributions (see Table A1). Historical morphological records restricted *L. annulatus* to the Southern Ocean and *L. radiosus* to southern South America (see Table A1). However, *L. radiosus* has recently been genetically identified in the Southern Ocean at Leclaire Rise (as Kerguelen in Vantomme et al. 2023). The presence of *Labidiaster* species in the Ross Sea has been historically unclear. While *L. radiosus* has never been observed in the Ross Sea, Dawson (1970) morphologically identified *L. annulatus* from this region; however, McKnight (1976) and Dearborn et al. (1991) later noted the absence of this species from the Ross Sea. This study provides the first genetic identification of *L. radiosus* at Discovery Bank, Herdman Bank, Heard Island, Admiralty Seamount, and Scott Island, and the first genetic confirmation of *L. radiosus* in the Ross Sea, resolving the historical uncertainty about the presence of *Labidiaster* in this region. Furthermore, this study provides the first identification of *L. annulatus* further north at Burdwood Bank and east of the Falkland Islands/Malvinas, north of the APF. This provides evidence that *Labidiaster* species both have a circum‐Antarctic distribution in the Southern Ocean.

Even though *L. annulatus* and *L. radiosus* co‐occur at some locations, their distributional ranges do not entirely overlap. These *Labidiaster* species co‐occur at locations around the Kerguelen Plateau, Scott Island and the Admiralty Seamount. Whether the presence of only a single *Labidiaster* species at some locations is an accurate representation of the benthos at that location or is due to under‐sampling is challenging to clarify. For example, *L. annulatus* and *L. radiosus* were both found in the Scotia Arc; however, only *L. radiosus* was sampled at Discovery and Herdman Banks. Eleven hauls were conducted at one station at Discovery Bank, ranging in depth from 379 to 762 m (Figure 1 and Table A2); however, being only a single station, the absence of *L. annulatus* from Discovery Bank may still reflect under‐sampling. Furthermore, the presence of both species at similar depths at Scott and Heard Islands indicates that they can co‐occur in habitats. These distributional patterns highlight the need for genetic analysis to better understand the evolutionary relationships and population connectivity between these *Labidiaster* species.

Phylogenetic analyses revealed that *L. annulatus* and *L. radiosus* form a sister‐species relationship. This relationship has been demonstrated by two distinct monophyletic clades with strong support from species delimitation analyses and clear separation within the COI haplotype network. While historical morphological studies have long recognized these as two distinct *Labidiaster* species (Dearborn et al. 1991; Fisher 1918, 1940; Réne Koehler 1923b; Koehler 1923a; Sladen 1889), the molecular data now confirm them as distinct species and reveal their close evolutionary relationship. This integration of morphological species recognition and phylogenetic analysis provides a foundation for examining the genetic mechanisms underlying their evolutionary divergence.

Mitochondrial DNA (mtDNA) has been successfully used for phylogenetic reconstruction and species‐level resolution for many Southern Ocean asteroids due to its high evolutionary rate, maternal inheritance, and small size. To complement the single‐parent inheritance perspective of mtDNA, a nuclear DNA (nuDNA) gene was analyzed using the intron 7 marker, which captures genetic history from both parents (see Foltz, Bolton, et al. 2007; Padial et al. 2010). While nuclear genes have longer coalescence times (29% that of COI in Foltz, Bolton, et al. 2007) and may lack resolution for closely related forcipulatacean sea stars (see Foltz, Nguyen, et al. 2007), they provide valuable historical signals (Foltz, Bolton, et al. 2007). The results showed that intron 7 did not separate the two *Labidiaster* species as clearly as mtDNA; however, both markers coincided in revealing significant genetic differentiation in *L. radiosus* populations north and south of the APF, and high gene flow among all *L. annulatus* locations. Genomic markers will likely be more powerful for revealing species boundaries as multiple loci inherited from both parents can be analyzed; this can also provide a higher resolution to understand divergence times and demographic histories.

### 4.2 When Did *L. radiosus* and *L. annulatus* Diverge, and Is This Timing Linked to Historical Changes?

The estimated divergence of *L. radiosus* and *L. annulatus* occurred during the Pliocene to early Pleistocene ~3.56 mya (HPD95: 5.25–2.15), which corresponds to the divergence time ranges (2–5 mya) of other closely‐related Southern Ocean sea stars (Asteroidea) species (within the genera *Cheiraster, Paralophaster* and *Odontaster* in Moreau et al. 2021). This suggests that environmental changes at this time drove the diversification of multiple Southern Ocean asteroid species. This time period is more recent than the suggested diversification of Southern Ocean forcipulatacean taxa (Eocene, Mah and Foltz 2011) coinciding with the timing (20–41 mya) of the ACC formation and subsequent cooling of the Southern Ocean waters (Ladant et al. 2014; Lagabrielle et al. 2009; Sijp et al. 2014). Since the formation of the ACC predates the divergence of the two *Labidiaster* species, it is unlikely that this process caused their divergence. Furthermore, the impact of repeated glacial–interglacial cycles over the last 5 million years has been suggested to be the main driver of the present asteroid diversity patterns (see Moreau et al. 2021, the ‘Antarctic diversity pump’ hypothesis in Clarke and Crame 1992, 1989). The repeated glacial–interglacial cycles dramatically shifted available habitat. During glacial periods, populations became isolated in refugia; during interglacial periods, populations expanded and may have reconnected. This repeated isolation‐reconnection pattern promoted rapid speciation (Clarke and Crame 1992, 1989), such may be the case for the divergence of *L. radiosus* and *L. annulatus*.

Evidence for a population expansion in both *L. annulatus* and *L. radiosus* was detected around the time of the LGM (~20,000 years ago). Post‐LGM population expansion signatures are also shared with Southern Ocean benthic echinoderms such as the sea urchin *S. neumayeri* (Díaz et al. 2018, 2011), the brittle star *O. victoriae* (Lau et al. 2021) and *Amphiura eugeniae* Ljungman, 1867 (Sands et al. 2024). For *L. radiosus*, only the COI Bayesian Skyline Plot indicated a population expansion around the time of the LGM, but the neutrality tests did not (exception of Tajima's *D* significant negative result for Discovery Bank and Ross Sea but not supported by Fu's Fs). During the LGM, grounded ice sheets may have displaced benthic invertebrates from the continental shelf; these could have dispersed and survived glacial cycles in sub‐Antarctic or deep‐sea refugia locations and subsequently expanded during interglacials (e.g., in Allcock and Strugnell 2012; Brey et al. 1996; e.g., brittle star *O. victoriae* in Lau, Wilson, et al. 2023).

The Bayesian skyline plots indicate relatively low effective population sizes (Ne) for both *Labidiaster* species, lower than initially expected for marine invertebrates but consistent with patterns in high‐fecundity species, which may be influenced by sweepstakes reproduction (e.g., Miller et al. 2018) and post‐LGM dynamics (see Lau et al. 2020). These Ne values are slightly below those of other Southern Ocean invertebrates, such as ~10^6^ for the sea urchin *S. neumayeri* (Díaz et al. 2018) and the limpet *Nacella concinna* Strebel, 1908 (González‐Wevar et al. 2013), yet align closely with estimates for Southern Ocean brittle star *Ophionotus hexactis* E. A. Smith, 1876 (Lau et al. 2021) and the Mediterranean species *Ophiocomina nigra* Abildgaard in O.F. Müller, 1789 (Leiva et al. 2023). Such low Ne likely reflects common demographic constraints in Antarctic benthic taxa, potentially resulting from isolation and glacial history.

For *L. annulatus*, evidence of population expansion is supported by the haplotype pattern (“star‐like” pattern explained in Allcock and Strugnell 2012), haplotypic and nucleotide diversities (Slatkin and Hudson 1991). This haplotype pattern has been shown in other widely‐dispersing Southern Ocean invertebrates such as the sea urchin *S. neumayeri* (Díaz et al. 2018, 2011), the nematode *Parbolasia corrugatus* McIntosh, 1876 (Thornhill et al. 2008), and the benthic shrimp *Chorismus antarcticus* Pfeffer, 1887 (Raupach et al. 2010). The high COI haplotypic and low nucleotide diversities detected at Bransfield Strait, Elephant Island, and Shag Rocks suggest that *L. annulatus* may have persisted at these locations in refugia during glacial cycles (Lau et al. 2021, 2020). Supporting this, Shag Rocks has been identified as a refugia location for other benthic invertebrates such as the octopus *Pareledone turqueti* Joubin, 1905 (Strugnell et al. 2012) and the western Antarctic Peninsula for sea spider *Nymphon australe* Hodgson, 1902 (Soler‐Membrives et al. 2017). Similar to *L. annulatus* , *L. radiosus* may have persisted during the LGM in refugia locations and subsequently expanded its population, dispersing to available habitats after the LGM. However, contrary to the star‐like haplotype pattern for *L. annulatus* , the haplotype pattern for *L. radiosus* indicates limited dispersal between populations north and south of the APF. This difference in haplotype patterns shows that these sister‐species have responded differently to the recent glacial–interglacial cycles.

### 4.3 APF as a Dynamic Biogeographical Barrier That Permits or Isolates Gene Flow in Different Species

The APF has been recognized as a significant north–south biogeographical barrier; however, *L. annulatus* exhibits genetic connectivity across the APF, unlike *L. radiosus* , which shows limited genetic connectivity across the APF. Signatures of dispersal across the APF for *L. annulatus* were supported by a shared haplotype between individuals within the Scotia Arc and north of the APF. Dispersal across the APF has been possible for other benthic invertebrates (Dietz et al. 2019; Galaska et al. 2017; Maroni et al. 2022; Moles et al. 2021; Moore et al. 2018). In contrast to *L. annulatus* , the APF appears to have limited dispersal for *L. radiosus* , with subclade divergence on either side of the APF dating back to ~2.08 mya (HPD95: 3.48–0.58). Similarly, other benthic invertebrate studies show limited gene flow across the APF (Galaska et al. 2017; Hunter and Halanych 2008; Leese et al. 2010; Majewski et al. 2021; Page and Linse 2002; Thompson 2008), indicating that the APF can limit dispersal as a permeable barrier. The APF has been identified to limit dispersal between the *L. radiosus* subclades; however, high dispersal within the subclades was indicated.

### 4.4 Dispersal of *L. radiosus* North of the APF

For *L. radiosus* north of the APF, high dispersal with limited genetic differentiation was found between the Strait of Magellan and Falkland Islands/Malvinas. The high dispersal between these locations is also seen for the near shore limpet, *Siphonaria lessonii* Blainville, 1827 (pelagic larvae) (Fernández Iriarte et al. 2020) and the sea star *G. antarctica* (pelagic larvae) (Moore et al. 2018). Dispersal between the Strait of Magellan and Falkland Islands/Malvinas coincides with the Humboldt Current System, Cape Horn Current and the Patagonian Coastal Current, flowing towards the Falkland Islands/Malvinas (see figure 1 in González‐Wevar et al. 2023). However, previous studies have also identified genetic isolation between the Strait of Magellan and Falkland Islands/Malvinas, such as the direct‐developing *Siphonaria* species (González‐Wevar et al. 2018) and the isopod *Serolis paradoxa* Fabricius, 1775 (also a direct‐developer) (Leese et al. 2008), and the patellogastropods *Nacella deaurata* Gmelin, 1791, *Nacella magellanica* Gmelin, 1791, *Nacella mytilina* Helbling, 1779 (pelagic larvae suggested, González‐Wevar et al. 2023, 2012). This difference in connectivity patterns between *L. radiosus* and other benthic invertebrates north of the APF suggests that dispersal does not only depend on reproductive modes but likely also depends on species‐specific tolerances and larval behaviors (e.g., Fraser et al. 2017), and this becomes even more apparent when examining *L. radiosus* across its entire Southern Ocean distribution range.

### 4.5 Contrasting Historical Migration Patterns Between *L. annulatus* and *L. radiosus*

Across the vast Southern Ocean, *L. annulatus* showed low genetic differentiation with high dispersal throughout time. Within the Scotia Sea and Antarctic Peninsula, high historical and contemporary migration rates were detected over large geographical distances (> 1000 km). However, different historical and contemporary migration patterns through time were revealed from *L. radiosus* across the Southern Ocean. While contemporary migration was detected between all locations, only one‐directional historical migration was detected from Heard Island to Discovery Bank, and from the Strait of Magellan to the Falkland Islands/Malvinas. Inferred migration patterns have changed through time for *L. radiosus*, with limited historical migration but contemporary migration now maintaining connectivity across the APF and Southern Ocean. This shows that dispersal patterns have been influenced through time and are likely due to species‐specific factors (e.g., tolerances and larval behaviors), as otherwise the dispersal of *L. annulatus* would have also been affected.

Significant isolation‐by‐distance and isolation‐by‐depth signatures were only detected in *L. radiosus* but not for *L. annulatus*. Isolation‐by‐distance has been identified for other Southern Ocean benthic invertebrates (Lau et al. 2024; Moore et al. 2018). Isolation‐by‐distance occurs in populations that have remained stable over time, where adjacent populations exhibit a higher connectivity that decreases with geographical distance (Wright 1943, e.g., Baird et al. 2012).

Even though significant isolation‐by‐distance was detected, this could have been driven by the genetic distance between the two *L. radiosus* subclades on either side of the APF. However, when the subclade north of the APF was removed from the analysis, significant isolation‐by‐distance was still detected in the subclade south of the APF. This indicates that genetic distance is affected by the geographical distance among locations. In comparison, high dispersal across the Southern Ocean for *L. annulatus* was revealed, with no isolation‐by‐distance and low genetic differentiation among populations. Similar patterns of low genetic differentiation among populations over a broad geographical range have been reported for other Southern Ocean benthic invertebrates such as the ribbon worm *Parbolasia corrugatus* McIntosh 1876 (Thornhill et al. 2008), shrimp *Chorismus antarcticus* Pfeffer 1887 (Raupach et al. 2010) and gastropod *Nacella concinna* Strebel 1908 (González‐Wevar et al. 2013; Hoffman et al. 2013). These contrasting IBD patterns between sister‐species suggest that *L. radiosus* and *L. annulatus* have different dispersal strategies.

**FIGURE A1:**
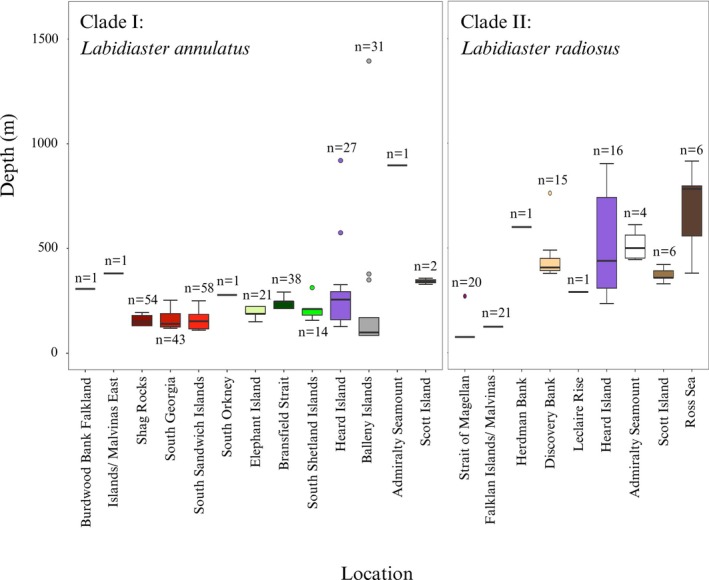
Showing collection depths (m) of *Labidiaster annulatus* and *Labidiaster radiosus* . The whiskers extend to most extreme points within 1.5 * interquartile range (IQR), the individual points show the outliers (> 1.5 * IQR), and the sample sizes (*n*) for each location are indicated above each whisker plot.

Both *Labidiaster* species display eurybathy (see Brey et al. 1996). *Labidiaster annulatus* was sampled between 85 and 1395 m and *L. radiosus* between 75 and 915 m in this study (Table A2 and Figure A1). Some genetic separation by depth has been indicated for other Southern Ocean benthic invertebrates (e.g., *Pareledone* sp. in Lau et al. 2024; Strugnell et al. 2017). However, the significant isolation‐by‐depth results for *L. radiosus* should be viewed with caution as specimens collected from the Strait of Magellan (mostly at 75 m) and Falkland Islands/Malvinas (124 m) were collected from shallower depths than specimens collected south of the APF (235–915 m, Table A2 and Figure A1). Furthermore, when the subclade north of the APF was removed from the analysis, no significant isolation‐by‐depth was detected in the subclade south of the APF. Therefore, it is difficult to determine whether the genetic distance is driven by the depth or due to a dispersal barrier between these subclades. The deep‐water Drake Passage, in between the continents, can reach ~4.2 km deep in some places (see Oldenhage et al. 2024; Riley et al. 2019), and changes in temperatures and current strength at the APF may further act as a dispersal barrier to gene flow, rather than depth or in addition to geographical distance (e.g., González‐Wevar et al. 2022, 2010, 2011, 2016, 2018, 2019, 2021; Poulin et al. 2014).

Unlike the circum‐Antarctic high genetic connectivity for *L. annulatus* populations, the dispersal pattern of *L. radiosus* suggests a circum‐Antarctic stepping‐stone pattern south of the APF (see Linse et al. 2007; Moore et al. 2018; Poulin et al. 2014). This is shown by increasing genetic differentiation and decreasing number of immigrants (contemporary migration) with increasing geographical distance across the Southern Ocean. A stepping‐stone dispersal pattern can aid gene flow across geographically distant locations, which would otherwise be unreachable using an organism's larval dispersal mode (see Linse et al. 2007; Moore et al. 2018; Poulin et al. 2014). Janosik et al. (2008) confirmed that *L. annulatus* in the Southern Ocean produces pelagic larvae using mtDNA (16Sr RNA) and suggested that these pelagic larvae may have been planktotrophic (feeding) larvae. While the contrasting genetic population structures between the two *Labidiaster* sister‐species in this study seemingly suggest different developmental modes, a sequence from a bipinnaria larval sample from the Ross Sea now confirms *L. radiosus* also produces planktotrophic (feeding) larvae. Larval ecology and behavioral idiosyncrasies may be responsible for the differing patterns between the two *Labidiaster* sister‐species. However, both *L. radiosus* and *L. annulatus* larvae were collected in the upper water column ( *L. radiosus* ~50 m, *L. annulatus* between 0 to 180 m), in early summer (November to December 2004) and during the same larval stage (bipinnaria, Mary Sewell, pers. comm., Heimeier et al. 2010; Janosik et al. 2008). The reason for these contrasting genetic population structures between the two *Labidiaster* sister‐species is enigmatic.

### 4.6 Shag Rocks as a Key Contemporary Sink Location for *L. annulatus*

Contemporary migration analyses and significant neutrality tests identify Shag Rocks as the largest sink location among all locations for *L. annulatus* . Sink populations cannot sustain themselves through local reproduction alone and require continuous immigration from source populations to persist (Pulliam 1988). Shag Rocks received a large number of immigrants from locations within the Scotia Sea and Antarctic Peninsula but supplied no emigrants to other populations. Shag Rocks shows high haplotype and nucleotide diversities, corroborating the contemporary migration result that immigrants originate from multiple locations and/or suggesting that the population is stable. Shag Rocks was also identified as a sink population for the benthic octopus *P. turqueti* (historical and contemporary migration in Lau et al. 2024). However, it is unknown whether the *L. annulatus* population at Shag Rocks will be maintained as the climate shifts, but identifying its contemporary source populations can inform conservation efforts by highlighting which populations require consideration.

### 4.7 Concluding Remarks

Despite the similarity in life history and circum‐Antarctic distributions, *L. radiosus* and *L. annulatus* have contrasting population structures. Genetic connectivity was maintained across the APF for *L. annulatus* but was limited between the *L. radiosus* subclades. Despite genetic population structure differences between the two *Labidiaster* sister‐species, suggesting alternative developmental modes, this study now establishes that *L. radiosus* similarly undergoes planktotrophic larval development. *Labidiaster radiosus* may produce larvae with additional limited dispersal capabilities or narrower environmental or climate change tolerances. Meanwhile, *L. annulatus* larvae may be better adapted for long‐distance dispersal, enabling the high connectivity to geographically far locations across the Southern Ocean and possibly explaining why the sister‐species show different dispersal patterns. Recent genetic evidence from the Southern Ocean now highlights contrasting genetic differentiation patterns between closely related Southern Ocean benthic octopodes (Strugnell et al. 2017), sea slugs *Doris* ‘*kergulenensis*’ (Maroni and Wilson 2022), and for other asteroid species (Vantomme et al. 2023) with similar life histories. These contrasting patterns challenge traditional assumptions by demonstrating that life history alone does not predict successful dispersal in the Southern Ocean, and that different species navigate Southern Ocean dispersal barriers in different ways. This highlights the unpredictable nature of dispersal for Southern Ocean benthic invertebrates, even between sister‐species.

## Author Contributions

**Nicola Rodewald:** conceptualization (equal), data curation (supporting), formal analysis (lead), funding acquisition (supporting), investigation (lead), methodology (lead), writing – original draft (lead), writing – review and editing (lead). **Nerida G. Wilson:** conceptualization (equal), data curation (lead), formal analysis (equal), funding acquisition (equal), investigation (equal), supervision (equal), writing – review and editing (equal). **Sally C. Y. Lau:** conceptualization (equal), formal analysis (equal), investigation (equal), supervision (equal), writing – review and editing (equal). **Jan M. Strugnell:** conceptualization (equal), formal analysis (equal), funding acquisition (equal), investigation (equal), supervision (equal), writing – review and editing (equal).

## Funding

This work was supported by the Competitive Research Training Grant from the College of Science and Engineering at James Cook University awarded to N.R. This work was also supported by ARC SRIEAS Grant SR200100005 Securing Antarctica's Environmental Future and the National Science Foundation Polar Programs (ANT‐1043749 to N.G.W.). This work contributes to delivering the Australian Antarctic Science Decadal Strategy.

## Conflicts of Interest

The authors declare no conflicts of interest.

## Supporting information


**Figure S1:** ece373450‐sup‐0001‐Supinfo.docx. *Labidiaster radiosus* early bipinnaria larva at 100× magnification (Mary Sewell, pers. comm.). Collected at Cape Hallett, Ross Sea on 3 December 2004 (GenBank: GU227094).
**Figure S2:** Maximum clade credibility tree showing median divergence time estimates on nodes for Labidiaster species as estimated from COI sequences. Dashed boxes show Labidiaster annulatus (Clade I) and Labidiaster radiosus (Clade II), and subclades within Labidiaster radiosus (Clade II) are labelled respectively. Purple boxes on node bars represent 95% height HPD. The scale bar represents branch lengths, and the scale axis indicates million years before present.
**Figure S3:** Extended Coalescent Bayesian skyline plot for Labidiaster annulatus showing effective population sizes (log(Ne)) across time (thousand years ago) for combined COI and Intron 7. The dashed black line represents the median and the grey shading on either side of the line represents 95% highest posterior density.
**Figure S4:** Extended Coalescent Bayesian skyline plot for Labidiaster radiosus showing effective population sizes (log(Ne)) across time (thousand years ago) for combined COI and Intron 7. The dashed black line represents the median and the grey shading on either side of the line represents 95% highest posterior density.
**Figure S5:** Relationship between genetic distance, represented by the pairwise genetic differentiation (FST) and the linearized FST (FST/(1‐FST)), and the geographical distances (km) among locations for Labidiaster annulatus COI (a, c) and intron 7 (b, d). The black line represents the linear regression line with its standard error shown by the grey shading above and below the black line. The linear equation, the coefficient of determination (r^2^) and its associated significance (*p*‐value) is shown in the top right of the plot. The *p*‐value is statistically significant at *p* < 0.05 and indicated by an asterisk (*). Locations for COI with *n* < 3 (Burdwood Bank, Falkland Islands/Malvinas East, South Orkney Islands, Admiralty Seamount, Scott Island) and for intron 7 with *n* < 6 (Burdwood Bank, Falkland Islands/Malvinas East, South Orkney Islands, Scott Island) were not used in the plot.
**Figure S6:** Relationship between genetic distance, represented by the pairwise genetic differentiation (FST) and the linearized FST (FST/(1‐FST)), and the geographical distances (km) among locations for Labidiaster radiosus COI (a, c) and intron 7 (b, d). The black line represents the linear regression line with its standard error shown by the grey shading above and below the black line. The linear equation, the coefficient of determination (R^2^) and its associated significance (*p*‐value) is shown in the top right of the plot. The *p*‐value is statistically significant at *p* < 0.05 and indicated by an asterisk (*). Locations for COI with *n* < 3 (Herdman Bank, Leclaire Rise) and for intron 7 with *n* < 6 (Herdman Bank, Leclaire Rise, Admiralty Seamount) were not used in the plot. Square indicates among locations north of the Antarctic Polar Front, triangles indicate between locations north and south of the Antarctic Polar Front, and circles indicate only among locations south of the Antarctic Polar Front.
**Table S1:** AMOVA results for L. annulatus locations. Locations within the sequence dataset COI with *n* < 3 (Burdwood Bank, Falkland Islands/Malvinas East, South Orkney Islands, Admiralty Seamount, Scott Island) and intron 7 with *n* < 6 (Burdwood Bank, Falkland Islands/Malvinas East, South Orkney Islands, Scott Island) were not included in the analysis. Labidiaster radiosus locations for COI with *n* < 3 (Herdman Bank, Leclaire Rise) and for intron 7 with *n* < 6 (Herdman Bank, Leclaire Rise, Admiralty Seamount) were not included in the AMOVA analysis. Significant *p*‐value (*p* < 0.05) is shown with an asterisk (*).
**Table S2:** Migrate‐n results for Labidiaster annulatus (a) showing location groupings used and (b) models implemented in migrate‐n ranking from highest to lowest probability calculated from BEZIER log(mL).
**Table S3:** Migrate‐n results for Labidiaster radiosus (a) showing location groupings used and (b) models implemented in migrate‐n ranking from highest to lowest probability calculated from BEZIER log(mL).

## Data Availability

The sequence data that support the findings of this study are openly available in GenBank (NCBI) at https://www.ncbi.nlm.nih.gov/genbank/ under GenBank numbers PZ015499 to PZ015859, PZ091454 to PZ091778 and PZ094402. Code used for the analyses can be found at https://github.com/NRodewald/labidiaster_sanger.

## Appendix A

**TABLE A1:** Morphologically identified Southern Ocean *Labidiaster annulatus* and southern South American *Labidiaster radiosus* distributions and recently identified *Labidiaster radiosus* extended distributions.

	References
***Labidiaster annulatus***	
*Distribution within the Southern Ocean*	
Type localities: Kerguelen Island, Heard Island	Sladen (1889), redescribed as *Labidastrella annulata* in Verrill (1914) and as *L. annulatus* in R. Koehler (1917), Arafura Sea (unlikely, see Fisher 1940)
Kerguelen Island, Heard Islands, Antarctic Archipelago, South Orkneys, South Sandwich Islands, South Georgia, Shag Rocks	Fisher (1940)
Antarctic Peninsula	A. H. Clark (1951)
Kerguelen Island	A. M. Clark (1962)
Ross Sea	Dawson (1970)
Weddell Sea	Voß (1988)
Balleny Islands	MacDiarmid and Stewart (2015)
South Georgia, South Sandwich Islands, Antarctic Peninsula	Dearborn et al. (1991)
South Shetland Islands	Presler and Figielska (1997)
Scotia Arc Islands	Ramos (1999)
Antarctic Peninsula	Manjón‐Cabeza et al. (2001), Morales et al. (2024)
Shag Rocks, South Georgia, South Sandwich Islands	Kim and Thurber (2007)
Elephant Island, Antarctic Peninsula	McClintock et al. (2011)
***Labidiaster radiosus***	
*Distribution within southern South America*	
Type locality: Patagonia	Lütken (1871), Studer (1876)
Chile	(Bell 1881, 94) as *Labidiaster lütkeni*, Martínez Salinas et al. (2018)
Patagonia, Strait of Magellan	Sladen (1889)
Punta Arenas, Tierra del Fuego	Perrier (1891)
southern South America	Fisher (1918)
Northern coast of Argentina, Falkland Islands	*Labidiaster radiosus* and *Labidiaster crassus* in Réne Koehler (1923b) see Fisher (1940)
Falkland Islands, Tierra del Fuego, Cape Tres Puntas	Fisher (1940)
Northern Chile	Madsen (1956)
southern South America, Falkland Plateau and as far north as the Brazilian Basin	Carrera‐Rodríguez and Tommasi (1977)
Argentinian Coast	Botto et al. (2006)
East coast of Patagonia	Mutschke and Ríos (2006)
Strait of Magellan	Andrade et al. (2016)
North Patagonia, Central Patagonia	Häussermann et al. (2021)
Falkland Islands, Strait of Magellan	Barboza et al. (2011)
*Distribution within the Southern Ocean*	
South Georgia, South Shetland Islands	Barboza et al. (2011)
Kerguelen Island, Antarctic Peninsula	Vantomme et al. (2023)

**TABLE A2:** Sample information and associated metadata.

Species	Museum ID	COI (GenBank/BOLD)	Intron 7 (GenBank)	Collection date	Location name	Location code	Latitude	Longitude
*Heliaster helianthus*		JX130054	—		—	—	—	—
*Labidiaster annulatus*		SOA594‐12	—	05‐Apr‐2006	South Georgia West	SG	−53.61103	−37.87679
*Labidiaster annulatus*	SIO‐BIC W1284	PZ094402	PZ091454	04‐May‐2005	Heard Island	HI	−53.2075	73.78467
*Labidiaster annulatus*		ASULB077‐20	—	27‐Nov‐2013	Kerguelen Island	KI	−48.408	69.074
*Labidiaster annulatus*		ASULB097‐20	—	05‐Dec‐2014	Kerguelen Island	KI	−49.174	69.427
*Labidiaster annulatus*		DQ077920	—	29‐Jan‐2002	Elephant Island	EI	−60.971976	−55.113288
*Labidiaster annulatus*		KERAS013‐20	—	05‐Dec‐2014	Kerguelen Island	KI	−49.174	69.427
*Labidiaster annulatus*	MNHN‐IE‐2011‐460	SOA013‐12	—	31‐Aug‐2010	Kerguelen Island	KI	−47.416696	66.8
*Labidiaster annulatus*	MNHN‐IE‐2011‐495	SOA036‐12	—	04‐Sep‐2010	Kerguelen Island	KI	−48.451192	70.606949
*Labidiaster annulatus*	MNHN‐IE‐2011‐556	SOA073‐12	—	05‐Sep‐2010	Kerguelen Island	KI	−47.600022	70.266677
*Labidiaster annulatus*	MNHN‐IE‐2011‐875	SOA216‐12	—	02‐Sep‐2010	Kerguelen Island	KI	−48.178835	68.604826
*Labidiaster annulatus*	NIWA 38237_N0005	PZ015499	PZ091455	04‐Mar‐2008	Scott Island: A, C32	SI	−67.83716667	−179.5603333
*Labidiaster annulatus*	NIWA 38591_N0010	PZ015500	PZ091456	04‐Mar‐2008	Scott Island: A, C32	SI	−67.78933333	−179.636
*Labidiaster annulatus*	NIWA 38611_N0019	PZ015501	—	13‐Mar‐2008	Admiralty Seamount: C24	AS	−67.168	171.179
*Labidiaster annulatus*	NIWA 84668_N0234	PZ015502	—	11‐Mar‐2006	Balleny Islands: Young Island	BI	−66.556833	162.569833
*Labidiaster annulatus*	NIWA 84668_N0235	PZ015503	—	11‐Mar‐2006	Balleny Islands: Young Island	BI	−66.556833	162.569833
*Labidiaster annulatus*	NIWA 84668_N0236	PZ015504	—	11‐Mar‐2006	Balleny Islands: Young Island	BI	−66.556833	162.569833
*Labidiaster annulatus*	NIWA 84668_N0237	PZ015505	PZ091457	11‐Mar‐2006	Balleny Islands: Young Island	BI	−66.556833	162.569833
*Labidiaster annulatus*	NIWA 84669_N0232	PZ015506	PZ091458	11‐Mar‐2006	Balleny Islands: Young Island	BI	−66.556833	162.569833
*Labidiaster annulatus*	NIWA 84669_N0233	PZ015507	PZ091459	11‐Mar‐2006	Balleny Islands: Young Island	BI	−66.556833	162.569833
*Labidiaster annulatus*	NIWA 27922	PZ015508	PZ091460	11‐Mar‐2006	Balleny Islands: Buckle Island	BI	−66.756167	163.060167
*Labidiaster annulatus*	NIWA 27925	1/NZEC730‐09	PZ091461	08‐Mar‐2006	Balleny Islands: Sturge Island	BI	−67.231333	164.7115
*Labidiaster annulatus*	NIWA 87589	PZ015509	PZ091462	05‐Mar‐2004	Balleny Islands: Young Island	BI	−66.216167	162.4415
*Labidiaster annulatus*	NIWA 87590	PZ015510	PZ091463	04‐Mar‐2004	Balleny Islands: S‐100	BI	−67.434833	163.883
*Labidiaster annulatus*	NIWA 87647	PZ015511	—	04‐Mar‐2004	Balleny Islands: Sturge Island	BI	−67.422167	163.916667
*Labidiaster annulatus*	NIWA 87662	PZ015512	PZ091464	05‐Mar‐2004	Balleny Islands: Young Island	BI	−66.675167	162.757
*Labidiaster annulatus*	QMT G144538	PZ015513	PZ091465	25‐Mar‐2023	Heard Island: Plateau West	HI	−52.338	73.037
*Labidiaster annulatus*	QMT G144570	PZ015514	—	17‐Mar‐2023	Heard Island: Gunnari Ridge	HI	−52.618	75.129
*Labidiaster annulatus*	QMT G144571	PZ015515	PZ091466	27‐Mar‐2023	Heard Island: Plateau West	HI	−52.199	73.233
*Labidiaster annulatus*	QMT G144572	PZ015516	—	27‐Mar‐2023	Heard Island: Plateau West	HI	−52.23	73.64
*Labidiaster annulatus*	QMT G144573	PZ015517	PZ091467	23‐Mar‐2023	Heard Island: Plateau South East	HI	−52.294	74.17
*Labidiaster annulatus*	QMT G144574	PZ015518	PZ091468	23‐Mar‐2023	Heard Island: Plateau South East	HI	−52.294	74.17
*Labidiaster annulatus*	QMT G144575	PZ015519	PZ091469	23‐Mar‐2023	Heard Island: Plateau South East	HI	−52.294	74.17
*Labidiaster annulatus*	QMT G144576	PZ015520	PZ091470	23‐Mar‐2023	Heard Island: Plateau South East	HI	−52.321	74.118
*Labidiaster annulatus*	QMT G144585	PZ015521	PZ091471	28‐Mar‐2023	Heard Island: Plateau North	HI	−51.185	72.783
*Labidiaster annulatus*	QMT G144586	PZ015522	PZ091472	27‐Mar‐2023	Heard Island: Plateau West	HI	−52.23	73.64
*Labidiaster annulatus*	QMT G144587	PZ015523	PZ091473	25‐Mar‐2023	Heard Island: Plateau West	HI	−52.338	73.037
*Labidiaster annulatus*	QMT G144588	PZ015524	PZ091474	25‐Mar‐2023	Heard Island: Plateau West	HI	−52.639	72.737
*Labidiaster annulatus*	QMT G144590	PZ015525	—	18‐Mar‐2023	Heard Island: Gunnari Ridge	HI	−52.469	75.098
*Labidiaster annulatus*	QMT G144591	PZ015526	—	18‐Mar‐2023	Heard Island: Gunnari Ridge	HI	−52.469	75.098
*Labidiaster annulatus*	QMT G144592	PZ015527	PZ091475	20‐Mar‐2023	Heard Island: Plateau South East	HI	−52.97	74.28
*Labidiaster annulatus*	SIO‐BIC E11509	PZ015528	PZ091476	29‐Sep‐2011	South Georgia East	SG	−55.05153	−35.3955
*Labidiaster annulatus*	SIO‐BIC E11510	PZ015529	PZ091477	29‐Sep‐2011	South Georgia East	SG	−55.05153	−35.3955
*Labidiaster annulatus*	SIO‐BIC E11511	PZ015530	PZ091478	29‐Sep‐2011	South Georgia East	SG	−55.05153	−35.3955
*Labidiaster annulatus*	SIO‐BIC E11512	PZ015531	PZ091479	29‐Sep‐2011	South Georgia East	SG	−55.05153	−35.3955
*Labidiaster annulatus*	SIO‐BIC E11513	PZ015532	PZ091480	29‐Sep‐2011	South Georgia East	SG	−55.05153	−35.3955
*Labidiaster annulatus*	SIO‐BIC W5569	PZ015533	PZ091481	29‐Sep‐2011	South Georgia East	SG	−55.03881	−35.44801
*Labidiaster annulatus*	SIO‐BIC W5570	PZ015534	PZ091482	29‐Sep‐2011	South Georgia East	SG	−55.03881	−35.44801
*Labidiaster annulatus*	SIO‐BIC W5572	PZ015535	PZ091483	29‐Sep‐2011	South Georgia East	SG	−55.03881	−35.44801
*Labidiaster annulatus*	SIO‐BIC E11514	PZ015536	—	29‐Sep‐2011	South Georgia East	SG	−55.08104	−35.1726
*Labidiaster annulatus*	SIO‐BIC E11515	PZ015537	PZ091484	04‐Oct‐2011	South Sandwich Islands: Visokoi Island	SSI	−56.70875	−27.04858
*Labidiaster annulatus*	SIO‐BIC E11516	PZ015538	PZ091485	04‐Oct‐2011	South Sandwich Islands: Visokoi Island	SSI	−56.70875	−27.04858
*Labidiaster annulatus*	SIO‐BIC E11517	PZ015539	PZ091486	04‐Oct‐2011	South Sandwich Islands: Visokoi Island	SSI	−56.70875	−27.04858
*Labidiaster annulatus*	SIO‐BIC E11518	PZ015540	PZ091487	04‐Oct‐2011	South Sandwich Islands: Visokoi Island	SSI	−56.70875	−27.04858
*Labidiaster annulatus*	SIO‐BIC E11519	PZ015541	PZ091488	04‐Oct‐2011	South Sandwich Islands: Visokoi Island	SSI	−56.70875	−27.04858
*Labidiaster annulatus*	SIO‐BIC E11520	PZ015542	PZ091489	04‐Oct‐2011	South Sandwich Islands: Visokoi Island	SSI	−56.70875	−27.04858
*Labidiaster annulatus*	SIO‐BIC E11521	PZ015543	PZ091490	04‐Oct‐2011	South Sandwich Islands: Visokoi Island	SSI	−56.70875	−27.04858
*Labidiaster annulatus*	SIO‐BIC E11522	PZ015544	PZ091491	04‐Oct‐2011	South Sandwich Islands: Visokoi Island	SSI	−56.70875	−27.04858
*Labidiaster annulatus*	SIO‐BIC E11523	PZ015545	PZ091492	04‐Oct‐2011	South Sandwich Islands: Visokoi Island	SSI	−56.70875	−27.04858
*Labidiaster annulatus*	SIO‐BIC E11524	PZ015546	PZ091493	05‐Oct‐2011	South Sandwich Islands: Visokoi Island	SSI	−56.70875	−27.04858
*Labidiaster annulatus*	SIO‐BIC E11525	PZ015547	PZ091494	05‐Oct‐2011	South Sandwich Islands: Visokoi Island	SSI	−56.70875	−27.04858
*Labidiaster annulatus*	SIO‐BIC E11526	PZ015548	PZ091495	05‐Oct‐2011	South Sandwich Islands: Montagu Island	SSI	−58.46814	−26.21807
*Labidiaster annulatus*	SIO‐BIC E11527	PZ015549	PZ091496	05‐Oct‐2011	South Sandwich Islands: Montagu Island	SSI	−58.46814	−26.21807
*Labidiaster annulatus*	SIO‐BIC W5574	PZ015550	—	05‐Oct‐2011	South Sandwich Islands: Montagu Island	SSI	−58.46573	−26.21316
*Labidiaster annulatus*	SIO‐BIC E11528	PZ015551	PZ091497	06‐Oct‐2011	South Sandwich Islands: Montagu Island	SSI	−58.37847	−26.28341
*Labidiaster annulatus*	SIO‐BIC E11529	PZ015552	PZ091498	06‐Oct‐2011	South Sandwich Islands: Montagu Island	SSI	−58.37847	−26.28341
*Labidiaster annulatus*	SIO‐BIC E11530	PZ015553	PZ091499	05‐Oct‐2011	South Sandwich Islands: Montagu Island	SSI	−58.37733	−26.28236
*Labidiaster annulatus*	SIO‐BIC E11531	PZ015554	PZ091500	05‐Oct‐2011	South Sandwich Islands: Montagu Island	SSI	−58.37733	−26.28236
*Labidiaster annulatus*	SIO‐BIC E11532	PZ015555	PZ091501	05‐Oct‐2011	South Sandwich Islands: Montagu Island	SSI	−58.37733	−26.28236
*Labidiaster annulatus*	SIO‐BIC E11533	PZ015556	PZ091502	05‐Oct‐2011	South Sandwich Islands: Montagu Island	SSI	−58.37733	−26.28236
*Labidiaster annulatus*	SIO‐BIC E11534	PZ015557	PZ091503	05‐Oct‐2011	South Sandwich Islands: Montagu Island	SSI	−58.37733	−26.28236
*Labidiaster annulatus*	SIO‐BIC E11535	PZ015558	PZ091504	05‐Oct‐2011	South Sandwich Islands: Montagu Island	SSI	−58.37733	−26.28236
*Labidiaster annulatus*	SIO‐BIC E11536	PZ015559	PZ091505	05‐Oct‐2011	South Sandwich Islands: Montagu Island	SSI	−58.37733	−26.28236
*Labidiaster annulatus*	SIO‐BIC E11537	PZ015560	PZ091506	05‐Oct‐2011	South Sandwich Islands: Montagu Island	SSI	−58.37733	−26.28236
*Labidiaster annulatus*	SIO‐BIC E11538	PZ015561	PZ091507	06‐Oct‐2011	South Sandwich Islands: Montagu Island	SSI	−58.37579	−26.28369
*Labidiaster annulatus*	SIO‐BIC E11539	PZ015562	PZ091508	06‐Oct‐2011	South Sandwich Islands: Montagu Island	SSI	−58.37579	−26.28369
*Labidiaster annulatus*	SIO‐BIC E11540	PZ015563	PZ091509	06‐Oct‐2011	South Sandwich Islands: Montagu Island	SSI	−58.37579	−26.28369
*Labidiaster annulatus*	SIO‐BIC E11541	PZ015564	PZ091510	03‐Oct‐2011	South Sandwich Islands: Visokoi Island	SSI	−56.72348	−27.03589
*Labidiaster annulatus*	SIO‐BIC E11542	PZ015565	PZ091511	03‐Oct‐2011	South Sandwich Islands: Visokoi Island	SSI	−56.72348	−27.03589
*Labidiaster annulatus*	SIO‐BIC E11543	PZ015566	PZ091512	03‐Oct‐2011	South Sandwich Islands: Visokoi Island	SSI	−56.72348	−27.03589
*Labidiaster annulatus*	SIO‐BIC E11544	PZ015567	PZ091513	03‐Oct‐2011	South Sandwich Islands: Visokoi Island	SSI	−56.72348	−27.03589
*Labidiaster annulatus*	SIO‐BIC E11545	PZ015568	PZ091514	03‐Oct‐2011	South Sandwich Islands: Visokoi Island	SSI	−56.72348	−27.03589
*Labidiaster annulatus*	SIO‐BIC E11546	PZ015569	PZ091515	03‐Oct‐2011	South Sandwich Islands: Visokoi Island	SSI	−56.72348	−27.03589
*Labidiaster annulatus*	SIO‐BIC E11547	PZ015570	PZ091516	03‐Oct‐2011	South Sandwich Islands: Visokoi Island	SSI	−56.72348	−27.03589
*Labidiaster annulatus*	SIO‐BIC E11548	PZ015571	PZ091517	05‐Oct‐2011	South Sandwich Islands: Montagu Island	SSI	−58.37733	−26.28236
*Labidiaster annulatus*	SIO‐BIC E11549	PZ015572	PZ091518	05‐Oct‐2011	South Sandwich Islands: Montagu Island	SSI	−58.37733	−26.28236
*Labidiaster annulatus*	SIO‐BIC E11550	PZ015573	PZ091519	22‐Oct‐2011	Elephant Island	EI	−61.17365	−54.19918
*Labidiaster annulatus*	SIO‐BIC E11551	PZ015574	PZ091520	22‐Oct‐2011	Elephant Island	EI	−61.17365	−54.19918
*Labidiaster annulatus*	SIO‐BIC E11552	PZ015575	PZ091521	22‐Oct‐2011	Elephant Island	EI	−61.17365	−54.19918
*Labidiaster annulatus*	SIO‐BIC E11553	PZ015576	PZ091522	22‐Oct‐2011	Elephant Island	EI	−61.17365	−54.19918
*Labidiaster annulatus*	SIO‐BIC E11554	PZ015577	PZ091523	22‐Oct‐2011	Elephant Island	EI	−61.17365	−54.19918
*Labidiaster annulatus*	SIO‐BIC E11555	PZ015578	PZ091524	22‐Oct‐2011	Elephant Island	EI	−61.17365	−54.19918
*Labidiaster annulatus*	SIO‐BIC E11556	PZ015579	PZ091525	22‐Oct‐2011	Elephant Island	EI	−61.17365	−54.19918
*Labidiaster annulatus*	SIO‐BIC E11557	PZ015580	PZ091526	22‐Oct‐2011	Elephant Island	EI	−61.17365	−54.19918
*Labidiaster annulatus*	SIO‐BIC E11558	PZ015581	PZ091527	22‐Oct‐2011	Elephant Island	EI	−61.17365	−54.19918
*Labidiaster annulatus*	SIO‐BIC E11559	PZ015582	PZ091528	22‐Oct‐2011	Elephant Island	EI	−61.17365	−54.19918
*Labidiaster annulatus*	SIO‐BIC E11560	PZ015583	PZ091529	22‐Oct‐2011	Elephant Island	EI	−61.20662	−54.25217
*Labidiaster annulatus*	SIO‐BIC E11561	PZ015584	PZ091530	22‐Oct‐2011	Elephant Island	EI	−61.20662	−54.25217
*Labidiaster annulatus*	SIO‐BIC E11562	PZ015585	PZ091531	22‐Oct‐2011	Elephant Island	EI	−61.20662	−54.25217
*Labidiaster annulatus*	SIO‐BIC E11563	PZ015586	PZ091532	22‐Oct‐2011	Elephant Island	EI	−61.20662	−54.25217
*Labidiaster annulatus*	SIO‐BIC E11564	PZ015587	PZ091533	22‐Oct‐2011	Elephant Island	EI	−61.20662	−54.25217
*Labidiaster annulatus*	SIO‐BIC E11565	PZ015588	PZ091534	22‐Oct‐2011	Elephant Island	EI	−61.20662	−54.25217
*Labidiaster annulatus*	SIO‐BIC E11566	PZ015589	PZ091535	22‐Oct‐2011	Elephant Island	EI	−61.20662	−54.25217
*Labidiaster annulatus*	SIO‐BIC E11567	PZ015590	PZ091536	22‐Oct‐2011	Elephant Island	EI	−61.20662	−54.25217
*Labidiaster annulatus*	SIO‐BIC E11568	PZ015591	PZ091537	22‐Oct‐2011	Elephant Island	EI	−61.20662	−54.25217
*Labidiaster annulatus*	SIO‐BIC E11569	PZ015592	PZ091538	22‐Oct‐2011	Elephant Island	EI	−61.20662	−54.25217
*Labidiaster annulatus*	SIO‐BIC E11573	PZ015593	PZ091539	20‐Oct‐2011	South Orkney	SO	−60.55086	−45.17611
*Labidiaster annulatus*	SIO‐BIC E6160	PZ015711	PZ091540	16‐Apr‐2013	South Georgia West	SG	−53.720617	−36.844467
*Labidiaster annulatus*	SIO‐BIC E11604	PZ015595	PZ091541	21‐Apr‐2013	Falkland Islands/Islas Malvinas East	FIE	−53.919117	−52.0992
*Labidiaster annulatus*	SIO‐BIC W5576	PZ015596	PZ091542	26‐Sep‐2011	South Georgia West	SG	−53.80054	−37.21886
*Labidiaster annulatus*	SIO‐BIC W5577	PZ015597	PZ091543	26‐Sep‐2011	South Georgia West	SG	−53.80054	−37.21886
*Labidiaster annulatus*	SIO‐BIC W5578	PZ015598	PZ091544	24‐Sep‐2011	Shag Rocks	SR	−53.7211	−41.46267
*Labidiaster annulatus*	SIO‐BIC W5579	PZ015599	PZ091545	24‐Sep‐2011	Shag Rocks	SR	−53.7211	−41.46267
*Labidiaster annulatus*	SIO‐BIC W5581	PZ015600	PZ091546	24‐Sep‐2011	Shag Rocks	SR	−53.7211	−41.46267
*Labidiaster annulatus*	SIO‐BIC W5582	PZ015601	PZ091547	24‐Sep‐2011	Shag Rocks	SR	−53.7211	−41.46267
*Labidiaster annulatus*	SIO‐BIC W5584	PZ015602	PZ091548	24‐Sep‐2011	Shag Rocks	SR	−53.72473	−41.46267
*Labidiaster annulatus*	SIO‐BIC W5586	PZ015603	PZ091549	24‐Sep‐2011	Shag Rocks	SR	−53.72473	−41.46267
*Labidiaster annulatus*	SIO‐BIC E11582	PZ015604	PZ091550	26‐Sep‐2011	South Georgia West	SG	−53.76698	−37.21713
*Labidiaster annulatus*	SIO‐BIC E11584	PZ015605	PZ091551	26‐Sep‐2011	South Georgia West	SG	−53.76698	−37.21713
*Labidiaster annulatus*	SIO‐BIC W5595	PZ015606	PZ091552	24‐Sep‐2011	Shag Rocks	SR	−53.72219	−41.46619
*Labidiaster annulatus*	SIO‐BIC W5587	PZ015607	PZ091553	24‐Sep‐2011	Shag Rocks	SR	−53.72219	−41.46619
*Labidiaster annulatus*	SIO‐BIC W5588	PZ015608	PZ091554	24‐Sep‐2011	Shag Rocks	SR	−53.72219	−41.46619
*Labidiaster annulatus*	SIO‐BIC W5589	PZ015609	PZ091555	24‐Sep‐2011	Shag Rocks	SR	−53.72219	−41.46619
*Labidiaster annulatus*	SIO‐BIC W5591	PZ015610	PZ091556	24‐Sep‐2011	Shag Rocks	SR	−53.72219	−41.46619
*Labidiaster annulatus*	SIO‐BIC W5593	PZ015611	PZ091557	24‐Sep‐2011	Shag Rocks	SR	−53.72219	−41.46619
*Labidiaster annulatus*	SIO‐BIC W5607	PZ015612	PZ091558	23‐Sep‐2011	Shag Rocks	SR	−53.53233	−41.62819
*Labidiaster annulatus*	SIO‐BIC W5608	PZ015613	PZ091559	23‐Sep‐2011	Shag Rocks	SR	−53.53233	−41.62819
*Labidiaster annulatus*	SIO‐BIC W5609	PZ015614	PZ091560	23‐Sep‐2011	Shag Rocks	SR	−53.53233	−41.62819
*Labidiaster annulatus*	SIO‐BIC W5597	PZ015615	PZ091561	23‐Sep‐2011	Shag Rocks	SR	−53.53233	−41.62819
*Labidiaster annulatus*	SIO‐BIC W5599	PZ015616	PZ091562	23‐Sep‐2011	Shag Rocks	SR	−53.53233	−41.62819
*Labidiaster annulatus*	SIO‐BIC W5600	PZ015617	PZ091563	23‐Sep‐2011	Shag Rocks	SR	−53.53233	−41.62819
*Labidiaster annulatus*	SIO‐BIC W5601	PZ015618	PZ091564	23‐Sep‐2011	Shag Rocks	SR	−53.53233	−41.62819
*Labidiaster annulatus*	SIO‐BIC W5603	PZ015619	PZ091565	23‐Sep‐2011	Shag Rocks	SR	−53.53233	−41.62819
*Labidiaster annulatus*	SIO‐BIC W5604	PZ015620	PZ091566	23‐Sep‐2011	Shag Rocks	SR	−53.53233	−41.62819
*Labidiaster annulatus*	SIO‐BIC W5605	PZ015621	PZ091567	23‐Sep‐2011	Shag Rocks	SR	−53.53233	−41.62819
*Labidiaster annulatus*	SIO‐BIC W5606	PZ015622	PZ091568	23‐Sep‐2011	Shag Rocks	SR	−53.53233	−41.62819
*Labidiaster annulatus*	SIO‐BIC W5610	PZ015623	PZ091569	22‐Sep‐2011	Shag Rocks	SR	−53.42398	−42.01774
*Labidiaster annulatus*	SIO‐BIC W5611	PZ015624	PZ091570	22‐Sep‐2011	Shag Rocks	SR	−53.42398	−42.01774
*Labidiaster annulatus*	SIO‐BIC W5613	PZ015625	PZ091571	22‐Sep‐2011	Shag Rocks	SR	−53.42398	−42.01774
*Labidiaster annulatus*	SIO‐BIC W5614	PZ015626	PZ091572	22‐Sep‐2011	Shag Rocks	SR	−53.42398	−42.01774
*Labidiaster annulatus*	SIO‐BIC W5615	PZ015627	PZ091573	22‐Sep‐2011	Shag Rocks	SR	−53.42398	−42.01774
*Labidiaster annulatus*	SIO‐BIC W5616	PZ015628	PZ091574	22‐Sep‐2011	Shag Rocks	SR	−53.42398	−42.01774
*Labidiaster annulatus*	SIO‐BIC W5617	PZ015629	PZ091575	22‐Sep‐2011	Shag Rocks	SR	−53.42398	−42.01774
*Labidiaster annulatus*	SIO‐BIC W5619	PZ015630	PZ091576	22‐Sep‐2011	Shag Rocks	SR	−53.42398	−42.01774
*Labidiaster annulatus*	SIO‐BIC W5620	PZ015631	PZ091577	22‐Sep‐2011	Shag Rocks	SR	−53.42398	−42.01774
*Labidiaster annulatus*	SIO‐BIC W5621	PZ015632	PZ091578	22‐Sep‐2011	Shag Rocks	SR	−53.42398	−42.01774
*Labidiaster annulatus*	SIO‐BIC W5622	PZ015633	PZ091579	22‐Sep‐2011	Shag Rocks	SR	−53.42398	−42.01774
*Labidiaster annulatus*	SIO‐BIC E11586	PZ015634	PZ091580	26‐Sep‐2011	South Georgia West	SG	−53.8069	−37.217
*Labidiaster annulatus*	SIO‐BIC E11587	PZ015635	PZ091581	26‐Sep‐2011	South Georgia West	SG	−53.8069	−37.217
*Labidiaster annulatus*	SIO‐BIC E11588	PZ015636	PZ091582	26‐Sep‐2011	South Georgia West	SG	−53.8069	−37.217
*Labidiaster annulatus*	SIO‐BIC E11589	PZ015637	PZ091583	26‐Sep‐2011	South Georgia West	SG	−53.8069	−37.217
*Labidiaster annulatus*	SIO‐BIC E11590	PZ015638	PZ091584	26‐Sep‐2011	South Georgia West	SG	−53.8069	−37.217
*Labidiaster annulatus*	SIO‐BIC E11591	PZ015639	PZ091585	31‐Oct‐2011	Burdwood Bank	BB	−54.66501	−61.23575
*Labidiaster annulatus*	SIO‐BIC W5623	PZ015640	PZ091586	28‐Oct‐2011	South Shetland Islands	SSH	−62.24668	−60.63124
*Labidiaster annulatus*	SIO‐BIC W5626	PZ015641	PZ091587	28‐Oct‐2011	South Shetland Islands	SSH	−62.24668	−60.63124
*Labidiaster annulatus*	SIO‐BIC W5627	PZ015642	PZ091588	28‐Oct‐2011	South Shetland Islands	SSH	−62.24668	−60.63124
*Labidiaster annulatus*	SIO‐BIC W5628	PZ015643	PZ091589	28‐Oct‐2011	South Shetland Islands	SSH	−62.24668	−60.63124
*Labidiaster annulatus*	SIO‐BIC W5629	PZ015644	PZ091590	28‐Oct‐2011	South Shetland Islands	SSH	−62.24668	−60.63124
*Labidiaster annulatus*	SIO‐BIC W5630	PZ015645	PZ091591	28‐Oct‐2011	South Shetland Islands	SSH	−62.24668	−60.63124
*Labidiaster annulatus*	SIO‐BIC W5631	PZ015646	PZ091592	28‐Oct‐2011	South Shetland Islands	SSH	−62.24668	−60.63124
*Labidiaster annulatus*	SIO‐BIC E11592	PZ015647	PZ091593	28‐Oct‐2011	South Shetland Islands	SSH	−62.1514	−60.65615
*Labidiaster annulatus*	SIO‐BIC E11594	PZ015648	PZ091594	27‐Oct‐2011	South Shetland Islands	SSH	−62.37028	−60.72834
*Labidiaster annulatus*	SIO‐BIC E11595	PZ015649	PZ091595	27‐Oct‐2011	South Shetland Islands	SSH	−62.33527	−60.77252
*Labidiaster annulatus*	SIO‐BIC E11596	PZ015650	PZ091596	27‐Oct‐2011	South Shetland Islands	SSH	−62.33527	−60.77252
*Labidiaster annulatus*	SIO‐BIC E11598	PZ015651	PZ091597	27‐Oct‐2011	South Shetland Islands	SSH	−62.33527	−60.77252
*Labidiaster annulatus*	SIO‐BIC W5632	PZ015652	PZ091598	25‐Oct‐2011	Bransfield Strait	BS	−63.34327	−59.91016
*Labidiaster annulatus*	SIO‐BIC W5633	PZ015653	PZ091599	25‐Oct‐2011	Bransfield Strait	BS	−63.34327	−59.91016
*Labidiaster annulatus*	SIO‐BIC W5634	PZ015654	PZ091600	25‐Oct‐2011	Bransfield Strait	BS	−63.34327	−59.91016
*Labidiaster annulatus*	SIO‐BIC W5635	PZ015655	PZ091601	25‐Oct‐2011	Bransfield Strait	BS	−63.34327	−59.91016
*Labidiaster annulatus*	SIO‐BIC W5636	PZ015656	PZ091602	25‐Oct‐2011	Bransfield Strait	BS	−63.34327	−59.91016
*Labidiaster annulatus*	SIO‐BIC W5638	PZ015657	PZ091603	25‐Oct‐2011	Bransfield Strait	BS	−63.34327	−59.91016
*Labidiaster annulatus*	SIO‐BIC W5639	PZ015658	PZ091604	25‐Oct‐2011	Bransfield Strait	BS	−63.34327	−59.91016
*Labidiaster annulatus*	SIO‐BIC W5640	PZ015659	PZ091605	25‐Oct‐2011	Bransfield Strait	BS	−63.34327	−59.91016
*Labidiaster annulatus*	SIO‐BIC W5641	PZ015660	PZ091606	25‐Oct‐2011	Bransfield Strait	BS	−63.34327	−59.91016
*Labidiaster annulatus*	SIO‐BIC W5645	PZ015661	PZ091607	25‐Oct‐2011	Bransfield Strait	BS	−63.34327	−59.91016
*Labidiaster annulatus*	SIO‐BIC W5646	PZ015662	PZ091608	25‐Oct‐2011	Bransfield Strait	BS	−63.34327	−59.91016
*Labidiaster annulatus*	SIO‐BIC W5647	PZ015663	PZ091609	25‐Oct‐2011	Bransfield Strait	BS	−63.34327	−59.91016
*Labidiaster annulatus*	SIO‐BIC W5648	PZ015664	PZ091610	25‐Oct‐2011	Bransfield Strait	BS	−63.34327	−59.91016
*Labidiaster annulatus*	SIO‐BIC W5649	PZ015665	PZ091611	25‐Oct‐2011	Bransfield Strait	BS	−63.34327	−59.91016
*Labidiaster annulatus*	SIO‐BIC E11599	PZ015666	PZ091612	24‐Oct‐2011	Bransfield Strait Mouth	BS	−62.75285	−57.32168
*Labidiaster annulatus*	SIO‐BIC W5650	PZ015667	PZ091613	24‐Oct‐2011	Bransfield Strait Mouth	BS	−62.86951	−57.21682
*Labidiaster annulatus*	SIO‐BIC W5653	PZ015668	PZ091614	24‐Oct‐2011	Bransfield Strait Mouth	BS	−62.86951	−57.21682
*Labidiaster annulatus*	SIO‐BIC W5654	PZ015669	PZ091615	25‐Oct‐2011	Bransfield Strait Mouth	BS	−62.86951	−57.21682
*Labidiaster annulatus*	SIO‐BIC W5655	PZ015670	PZ091616	24‐Oct‐2011	Bransfield Strait Mouth	BS	−62.86951	−57.21682
*Labidiaster annulatus*	SIO‐BIC W5656	PZ015671	PZ091617	26‐Oct‐2011	Bransfield Strait Mouth	BS	−62.86951	−57.21682
*Labidiaster annulatus*	SIO‐BIC W5657	PZ015672	PZ091618	24‐Oct‐2011	Bransfield Strait Mouth	BS	−62.86951	−57.21682
*Labidiaster annulatus*	SIO‐BIC W5658	PZ015673	PZ091619	27‐Oct‐2011	Bransfield Strait Mouth	BS	−62.86951	−57.21682
*Labidiaster annulatus*	SIO‐BIC W5660	PZ015674	PZ091620	24‐Oct‐2011	Bransfield Strait Mouth	BS	−62.86951	−57.21682
*Labidiaster annulatus*	SIO‐BIC W5661	PZ015675	PZ091621	28‐Oct‐2011	Bransfield Strait Mouth	BS	−62.86951	−57.21682
*Labidiaster annulatus*	SIO‐BIC W5662	PZ015676	PZ091622	24‐Oct‐2011	Bransfield Strait Mouth	BS	−62.86951	−57.21682
*Labidiaster annulatus*	SIO‐BIC W5663	PZ015677	PZ091623	29‐Oct‐2011	Bransfield Strait Mouth	BS	−62.86951	−57.21682
*Labidiaster annulatus*	SIO‐BIC W5664	PZ015678	PZ091624	24‐Oct‐2011	Bransfield Strait Mouth	BS	−62.86951	−57.21682
*Labidiaster annulatus*	SIO‐BIC W5665	PZ015679	PZ091625	30‐Oct‐2011	Bransfield Strait Mouth	BS	−62.86951	−57.21682
*Labidiaster annulatus*	SIO‐BIC W5666	PZ015680	PZ091626	24‐Oct‐2011	Bransfield Strait Mouth	BS	−62.86951	−57.21682
*Labidiaster annulatus*	SIO‐BIC E5058	PZ015681	PZ091627	24‐Oct‐2011	Bransfield Strait Mouth	BS	−62.86951	−57.21682
*Labidiaster annulatus*	SIO‐BIC E11600	PZ015682	—	24‐Oct‐2011	Bransfield Strait Mouth	BS	−62.86951	−57.21682
*Labidiaster annulatus*	SIO‐BIC E11601	PZ015683	PZ091628	23‐Oct‐2011	Elephant Island	EI	−61.27566	−55.77709
*Labidiaster annulatus*	SIO‐BIC E7111	MN249443/GBSP16594‐19	PZ091666	16‐Apr‐2013	South Georgia West	SG	−53.7151	−36.8357
*Labidiaster annulatus*	SIO‐BIC E4048	PZ015684	PZ091630	23‐Sep‐2011	Shag Rocks	SR	−53.53233	−41.62819
*Labidiaster annulatus*	SIO‐BIC E4756	PZ015685	PZ091631	10‐Jul‐2011	South Sandwich Islands	SSI	−59.39364	−27.32269
*Labidiaster annulatus*	SIO‐BIC E5024	PZ015686	PZ091632	22‐Sep‐2011	Shag Rocks	SR	−53.42398	−42.01774
*Labidiaster annulatus*	SIO‐BIC E5025	PZ015687	PZ091633	23‐Sep‐2011	Shag Rocks	SR	−53.53233	−41.62819
*Labidiaster annulatus*	SIO‐BIC E5026	PZ015688	PZ091634	29‐Sep‐2011	South Georgia	SG	−55.08104	−35.1726
*Labidiaster annulatus*	SIO‐BIC E5027	PZ015689	PZ091635	26‐Sep‐2011	South Georgia	SG	−53.80054	−37.21886
*Labidiaster annulatus*	SIO‐BIC E5031	PZ015690	PZ091636	23‐Sep‐2011	Shag Rocks	SR	−53.53233	−41.62819
*Labidiaster annulatus*	SIO‐BIC E5033	PZ015691	PZ091637	24‐Sep‐2011	Shag Rocks	SR	−53.7211	−41.46267
*Labidiaster annulatus*	SIO‐BIC E5034	PZ015692	PZ091638	22‐Sep‐2011	Shag Rocks	SR	−53.42398	−42.01774
*Labidiaster annulatus*	SIO‐BIC E5035	PZ015693	PZ091639	04‐Oct‐2011	South Sandwich Islands: Southern Thule Islands	SSI	−56.70875	−27.04858
*Labidiaster annulatus*	SIO‐BIC E5036	PZ015694	PZ091640	23‐Sep‐2011	Shag Rocks	SR	−53.53233	−41.62819
*Labidiaster annulatus*	SIO‐BIC E5037	PZ015695	PZ091641	22‐Sep‐2011	Shag Rocks	SR	−53.42398	−42.01774
*Labidiaster annulatus*	SIO‐BIC E5038	PZ015696	PZ091642	29‐Sep‐2011	South Georgia East	SG	−55.05153	−35.3955
*Labidiaster annulatus*	SIO‐BIC E5043	PZ015697	PZ091643	04‐Oct‐2011	South Sandwich Islands: Southern Thule Islands	SSI	−56.70875	−27.04858
*Labidiaster annulatus*	SIO‐BIC E5045	PZ015698	PZ091644	23‐Sep‐2011	Shag Rocks	SR	−53.53233	−41.62819
*Labidiaster annulatus*	SIO‐BIC E5046	PZ015699	PZ091645	29‐Sep‐2011	South Georgia East	SG	−55.03881	−35.44801
*Labidiaster annulatus*	SIO‐BIC E5047	PZ015700	PZ091646	23‐Sep‐2011	Shag Rocks	SR	−53.53233	−41.62819
*Labidiaster annulatus*	SIO‐BIC E5049	PZ015701	PZ091647	29‐Sep‐2011	South Georgia East	SG	−55.03881	−35.44801
*Labidiaster annulatus*	SIO‐BIC E5050	PZ015702	PZ091648	29‐Sep‐2011	South Georgia East	SG	−55.03881	−35.44801
*Labidiaster annulatus*	SIO‐BIC E5052	PZ015703	PZ091649	24‐Sep‐2011	Shag Rocks	SR	−53.7211	−41.46267
*Labidiaster annulatus*	SIO‐BIC E5055	PZ015704	PZ091650	04‐Oct‐2011	South Sandwich Islands: Southern Thule Islands	SSI	−56.70875	−27.04858
*Labidiaster annulatus*	SIO‐BIC E5056	PZ015705	PZ091651	29‐Sep‐2011	South Georgia East	SG	−55.03881	−35.44801
*Labidiaster annulatus*	SIO‐BIC E5057	PZ015706	PZ091652	24‐Oct‐2011	Bransfield Strait Mouth	BS	−62.86951	−57.21682
*Labidiaster annulatus*	SIO‐BIC W5652	PZ015707	PZ091629	24‐Oct‐2011	Bransfield Strait Mouth	BS	−62.86951	−57.21682
*Labidiaster annulatus*	SIO‐BIC E5059	PZ015708	PZ091653	29‐Sep‐2011	South Georgia East	SG	−55.05153	−35.3955
*Labidiaster annulatus*	SIO‐BIC E5063	PZ015709	PZ091654	04‐Oct‐2011	South Sandwich Islands: Southern Thule Islands	SSI	−56.70875	−27.04858
*Labidiaster annulatus*	SIO‐BIC E5068	PZ015710	PZ091655	26‐Sep‐2011	South Georgia West	SG	−53.80054	−37.21886
*Labidiaster annulatus*	SIO‐BIC E6425	PZ015594	PZ091656	16‐Apr‐2013	South Georgia West	SG	−53.720617	−36.844467
*Labidiaster annulatus*	SIO‐BIC E6431	PZ015712	PZ091657	16‐Apr‐2013	South Georgia West	SG	−53.619533	−37.35035
*Labidiaster annulatus*	SIO‐BIC E6432	PZ015713	PZ091658	16‐Apr‐2013	South Georgia West	SG	−53.619533	−37.35035
*Labidiaster annulatus*	SIO‐BIC E6433	PZ015714	PZ091659	16‐Apr‐2013	Shag Rocks	SR	−53.619533	−37.35035
*Labidiaster annulatus*	SIO‐BIC E6434	PZ015715	PZ091660	16‐Apr‐2013	South Georgia West	SG	−53.619533	−37.35035
*Labidiaster annulatus*	SIO‐BIC E6441	PZ015716	PZ091661	16‐Apr‐2013	South Georgia West	SG	−53.720617	−36.844467
*Labidiaster annulatus*	SIO‐BIC E6444	PZ015717	PZ091662	16‐Apr‐2013	South Georgia West	SG	−53.619533	−37.35035
*Labidiaster annulatus*	SIO‐BIC E6445	PZ015718	PZ091663	16‐Apr‐2013	South Georgia West	SG	−53.619533	−37.35035
*Labidiaster annulatus*	SIO‐BIC E6447	PZ015719	PZ091664	16‐Apr‐2013	South Georgia West	SG	−53.715167	−36.83575
*Labidiaster annulatus*	SIO‐BIC E6737	PZ015720	PZ091665	03‐Oct‐2011	South Sandwich Islands: Candlemas Island	SSI	−57.03507	−26.73767
*Labidiaster annulatus*	SIO‐BIC W1262	PZ015721	PZ091667	04‐May‐2005	Heard Island	HI	−53.10367	72.905
*Labidiaster annulatus*	SIO‐BIC W1264	PZ015722	—	04‐May‐2005	Heard Island	HI	−53.2075	73.78467
*Labidiaster annulatus*	SIO‐BIC W1269	PZ015723	—	05‐May‐2005	Heard Island	HI	−53.09	74.10667
*Labidiaster annulatus*	SIO‐BIC W1278	PZ015724	—	04‐May‐2005	Heard Island	HI	−53.2075	73.78467
*Labidiaster annulatus*	SIO‐BIC W1279	PZ015725	—	04‐May‐2005	Heard Island	HI	−53.2075	73.78467
*Labidiaster annulatus*	SIO‐BIC W1280	PZ015726	—	04‐May‐2005	Heard Island	HI	−53.2075	73.78467
*Labidiaster annulatus*	SIO‐BIC W1281	PZ015727	—	04‐May‐2005	Heard Island	HI	−53.2075	73.78467
*Labidiaster annulatus*	SIO‐BIC W1285	PZ015728	PZ091668	04‐May‐2005	Heard Island	HI	−53.2075	73.78467
*Labidiaster annulatus*	SIO‐BIC W1286	PZ015729	PZ091669	04‐May‐2005	Heard Island	HI	−53.2075	73.78467
*Labidiaster annulatus*	SIO‐BIC W1287	PZ015730	—	04‐May‐2005	Heard Island	HI	−53.2075	73.78467
*Labidiaster annulatus*	SIO‐BIC W1362	PZ015731	—	01‐Jun‐2006	Heard Island: Evitas Patch	HI	−53.055	74.70167
*Labidiaster annulatus*	SIO‐BIC W5567	PZ015732	PZ091670	29‐Sep‐2011	South Georgia East	SG	−55.03881	−35.44801
*Labidiaster annulatus*	SIO‐BIC W5568	PZ015733	PZ091671	29‐Sep‐2011	South Georgia East	SG	−55.03881	−35.44801
*Labidiaster annulatus*	SIO‐BIC W5571	PZ015734	PZ091672	29‐Sep‐2011	South Georgia East	SG	−55.03881	−35.44801
*Labidiaster annulatus*	SIO‐BIC W5573	PZ015735	PZ091673	29‐Sep‐2011	South Georgia East	SG	−55.03881	−35.44801
*Labidiaster annulatus*	SIO‐BIC W5575	PZ015736	PZ091674	26‐Sep‐2011	South Georgia West	SG	−53.80054	−37.21886
*Labidiaster annulatus*	SIO‐BIC W5580	PZ015737	PZ091675	24‐Sep‐2011	Shag Rocks	SR	−53.7211	−41.46267
*Labidiaster annulatus*	SIO‐BIC W5583	PZ015738	PZ091676	24‐Sep‐2011	Shag Rocks	SR	−53.7211	−41.46267
*Labidiaster annulatus*	SIO‐BIC W5585	PZ015739	PZ091677	24‐Sep‐2011	Shag Rocks	SR	−53.72473	−41.46267
*Labidiaster annulatus*	SIO‐BIC W5590	PZ015740	PZ091678	24‐Sep‐2011	Shag Rocks	SR	−53.72219	−41.46619
*Labidiaster annulatus*	SIO‐BIC W5592	PZ015741	PZ091679	24‐Sep‐2011	Shag Rocks	SR	−53.72219	−41.46619
*Labidiaster annulatus*	SIO‐BIC W5596	PZ015742	PZ091680	23‐Sep‐2011	Shag Rocks	SR	−53.53233	−41.62819
*Labidiaster annulatus*	SIO‐BIC W5612	PZ015743	PZ091681	22‐Sep‐2011	Shag Rocks	SR	−53.42398	−42.01774
*Labidiaster annulatus*	SIO‐BIC W5618	PZ015744	PZ091682	22‐Sep‐2011	Shag Rocks	SR	−53.42398	−42.01774
*Labidiaster annulatus*	SIO‐BIC W5624	PZ015745	PZ091683	28‐Oct‐2011	South Shetland Islands	SSH	−62.24668	−60.63124
*Labidiaster annulatus*	SIO‐BIC W5625	PZ015746	—	28‐Oct‐2011	South Shetland Islands	SSH	−62.24668	−60.63124
*Labidiaster annulatus*	SIO‐BIC W5637	PZ015747	PZ091684	25‐Oct‐2011	Bransfield Strait	BS	−63.34327	−59.91016
*Labidiaster annulatus*	SIO‐BIC W5643	PZ015748	PZ091685	25‐Oct‐2011	Bransfield Strait	BS	−63.34327	−59.91016
*Labidiaster annulatus*	SIO‐BIC W5644	PZ015749	PZ091686	25‐Oct‐2011	Bransfield Strait	BS	−63.34327	−59.91016
*Labidiaster annulatus*	SIO‐BIC W5651	PZ015750	—	24‐Oct‐2011	Bransfield Strait Mouth	BS	−62.86951	−57.21682
*Labidiaster annulatus*	SIO‐BIC W5659	PZ015751	—	24‐Oct‐2011	Bransfield Strait Mouth	BS	−62.86951	−57.21682
*Labidiaster annulatus*	SIO‐BIC W5667	PZ015752	PZ091687	16‐Apr‐2013	South Georgia West	SG	−53.715167	−36.83575
*Labidiaster annulatus*	WAM Z44250	PZ015753	PZ091688	02‐Mar‐2017	Balleny Islands: Young Island	BI	−66.1742	162.2029
*Labidiaster annulatus*	WAM Z44547	—	PZ091689	03‐Jul‐2017	South Sandwich Islands: Candlemas Island	SSI	−57.1489	−26.7546
*Labidiaster annulatus*	WAM Z44551	—	PZ091690	03‐Jul‐2017	South Sandwich Islands: Candlemas Island	SSI	−57.1501	−26.749
*Labidiaster annulatus*	WAM Z44572	PZ015754	PZ091691	03‐Jul‐2017	South Sandwich Islands: Candlemas Island	SSI	−57.1966	−26.7953
*Labidiaster annulatus*	WAM Z44573	PZ015755	PZ091692	03‐Jul‐2017	South Sandwich Islands: Candlemas Island	SSI	−57.1966	−26.7953
*Labidiaster annulatus*	WAM Z44604	PZ015756	PZ091693	03‐Aug‐2017	South Sandwich Islands: Southern Thule Islands	SSI	−59.4709	−27.2825
*Labidiaster annulatus*	WAM Z44734	PZ015757	PZ091694	03‐Jul‐2017	South Sandwich Islands: Candlemas Island	SSI	−57.1966	−26.7953
*Labidiaster annulatus*	WAM Z44735	PZ015758	PZ091695	03‐Jul‐2017	South Sandwich Islands: Candlemas Island	SSI	−57.1966	−26.7953
*Labidiaster annulatus*	WAM Z44736	PZ015759	PZ091696	03‐Jul‐2017	South Sandwich Islands: Candlemas Island	SSI	−57.1966	−26.7953
*Labidiaster annulatus*	WAM Z44737	PZ015760	PZ091697	03‐Jul‐2017	South Sandwich Islands: Candlemas Island	SSI	−57.1966	−26.7953
*Labidiaster annulatus*	WAM Z44846	PZ015761	PZ091698	03‐Jul‐2017	South Sandwich Islands: Candlemas Island	SSI	−57.1966	−26.7953
*Labidiaster annulatus*	WAM Z44847	PZ015762	PZ091699	03‐Jul‐2017	South Sandwich Islands: Candlemas Island	SSI	−57.1966	−26.7953
*Labidiaster annulatus*	WAM Z44848	PZ015763	PZ091700	03‐Jul‐2017	South Sandwich Islands: Candlemas Island	SSI	−57.1966	−26.7953
*Labidiaster annulatus*	WAM Z44855	PZ015764	PZ091701	03‐Jul‐2017	South Sandwich Islands: Candlemas Island	SSI	−57.1489	−26.7546
*Labidiaster annulatus*	WAM Z44856	PZ015765	PZ091702	03‐Jul‐2017	South Sandwich Islands: Candlemas Island	SSI	−57.1489	−26.7546
*Labidiaster annulatus*	WAM Z44857	PZ015766	PZ091703	03‐Jul‐2017	South Sandwich Islands: Candlemas Island	SSI	−57.1489	−26.7546
*Labidiaster annulatus*	WAM Z44858	PZ015767	PZ091704	03‐Jul‐2017	South Sandwich Islands: Candlemas Island	SSI	−57.1489	−26.7546
*Labidiaster annulatus*	WAM Z44859	PZ015768	—	03‐Jul‐2017	South Sandwich Islands: Candlemas Island	SSI	−57.1489	−26.7546
*Labidiaster annulatus*	WAM Z88479	PZ015769	PZ091705	03‐Jul‐2017	South Sandwich Islands: Candlemas Island	SSI	−57.1489	−26.7546
*Labidiaster radiosus*		GU227094/GBMIN880‐12	—	03‐Dec‐2004	Ross Sea: Cape Hellett	RS	−72.306873	170.188177
*Labidiaster radiosus*	NIWA 38227_N0006	PZ015770	PZ091706	04‐Mar‐2008	Scott Island: A, C32	SI	−67.83716667	−179.5603333
*Labidiaster radiosus*	NIWA 38237_N0004	PZ015771	PZ091707	05‐Mar‐2008	Scott Island: A, C33	SI	−67.855	−179.6136667
*Labidiaster radiosus*	NIWA 38270_N0009	PZ015772	PZ091708	04‐Mar‐2008	Scott Island: A, C34	SI	−67.78933333	−179.636
*Labidiaster radiosus*	NIWA 38318_N0012	PZ015773	—	04‐Mar‐2008	Scott Island: A, C35	SI	−67.85433333	−179.6445
*Labidiaster radiosus*	NIWA 38401_N0003	PZ015774	PZ091709	04‐Mar‐2008	Scott Island: A, C36	SI	−67.83716667	−179.5603333
*Labidiaster radiosus*	NIWA 38542_N0007	PZ015775	PZ091710	04‐Mar‐2008	Scott Island: A, C37	SI	−67.83716667	−179.5603333
*Labidiaster radiosus*	NIWA 104582	PZ015776	PZ091711	19‐Feb‐2015	Ross Sea	RS	−72.2385	174.6918333
*Labidiaster radiosus*	NIWA 140065	PZ015777	PZ091712	24‐Jan‐2019	Ross Sea: Iselin Bank	RS	−71.9095	−176.9033333
*Labidiaster radiosus*	NIWA 140163	PZ015778	—	30‐Jan‐2019	Ross Sea: Iselin Bank	RS	−74.4145	−176.7825
*Labidiaster radiosus*	NIWA 37284	PZ015779	PZ091713	21‐Feb‐2008	Ross Sea: C16	RS	−72.32883333	175.4708333
*Labidiaster radiosus*	NIWA 38784	PZ015780	PZ091714	10‐Mar‐2008	Admiralty Seamount: C24	AS	−66.9925	170.877
*Labidiaster radiosus*	NIWA 38832	PZ015781	PZ091715	10‐Mar‐2008	Admiralty Seamount: C25	AS	−66.99383333	170.88
*Labidiaster radiosus*	NIWA 38946	PZ015782	—	11‐Mar‐2008	Admiralty Seamount: C26	AS	−67.1215	170.9371667
*Labidiaster radiosus*	NIWA 39247	PZ015783	—	14‐Mar‐2008	Admiralty Seamount: C27	AS	−67.1705	171.1265
*Labidiaster radiosus*	NIWA 87588	PZ015784	PZ091716	27‐Feb‐2004	Ross Sea	RS	−71.493668	171.604172
*Labidiaster radiosus*	NIWA 87667	PZ015785	PZ091717	02‐Mar‐2004	Ross Sea	RS	−71.696667	172.073667
*Labidiaster radiosus*	QMT G144549	PZ015786	PZ091718	08‐Apr‐2023	Heard Island: Evitas 2	HI	−52.97	74.564
*Labidiaster radiosus*	QMT G144550	PZ015787	PZ091719	08‐Apr‐2023	Heard Island: Evitas 2	HI	−52.97	74.564
*Labidiaster radiosus*	QMT G144577	PZ015788	PZ091720	30‐Mar‐2023	Heard Island: Plateau Deep North East	HI	−51.085	75.331
*Labidiaster radiosus*	QMT G144589	PZ015789	PZ091721	06‐Apr‐2023	Heard Island: Evitas 2	HI	−52.997	74.582
*Labidiaster radiosus*	QMT G144593	PZ015790	PZ091722	20‐Mar‐2023	Heard Island: Plateau South East	HI	−52.97	74.28
*Labidiaster radiosus*	QMT G144594	PZ015791	PZ091723	20‐Mar‐2023	Heard Island: Plateau South East	HI	−52.97	74.28
*Labidiaster radiosus*	SIO‐BIC E11570	PZ015792	PZ091724	16‐Oct‐2011	Discovery Bank	DB	−60.13761	−34.86073
*Labidiaster radiosus*	SIO‐BIC E11571	PZ015793	PZ091725	16‐Oct‐2011	Discovery Bank	DB	−60.13761	−34.86073
*Labidiaster radiosus*	SIO‐BIC E11572	PZ015794	PZ091726	16‐Oct‐2011	Discovery Bank	DB	−60.14714	−34.87401
*Labidiaster radiosus*	SIO‐BIC W1684	PZ015795	—	16‐Oct‐2011	Discovery Bank	DB	−60.13761	−34.86073
*Labidiaster radiosus*	SIO‐BIC W1685	PZ015796	PZ091727	15‐Oct‐2011	Discovery Bank	DB	−60.1	−34.86431
*Labidiaster radiosus*	SIO‐BIC E11575	PZ015797	PZ091728	16‐Oct‐2011	Discovery Bank	DB	−60.12849	−34.94089
*Labidiaster radiosus*	SIO‐BIC E11576	PZ015798	PZ091729	16‐Oct‐2011	Discovery Bank	DB	−60.12849	−34.94089
*Labidiaster radiosus*	SIO‐BIC E11577	PZ015799	PZ091730	16‐Oct‐2011	Discovery Bank	DB	−60.13791	−34.88559
*Labidiaster radiosus*	SIO‐BIC E11578	PZ015800	PZ091731	15‐Oct‐2011	Discovery Bank	DB	−60.12743	−34.90311
*Labidiaster radiosus*	SIO‐BIC E11579	PZ015801	PZ091732	15‐Oct‐2011	Discovery Bank	DB	−60.12743	−34.90311
*Labidiaster radiosus*	SIO‐BIC E11580	PZ015802	PZ091733	15‐Oct‐2011	Discovery Bank	DB	−60.12743	−34.90311
*Labidiaster radiosus*	SIO‐BIC E11581	PZ015803	PZ091734	15‐Oct‐2011	Discovery Bank	DB	−60.10026	−34.89993
*Labidiaster radiosus*	SIO‐BIC E6435	PZ015804	PZ091735	10‐Apr‐2013	Strait of Magellan	SoM	−53.60225	−70.245667
*Labidiaster radiosus*	SIO‐BIC E6466	PZ015805	PZ091736	10‐Apr‐2013	Strait of Magellan	SoM	−53.509117	−70.406417
*Labidiaster radiosus*	SIO‐BIC E6514	PZ015806	PZ091737	10‐Apr‐2013	Strait of Magellan	SoM	−53.509117	−70.406417
*Labidiaster radiosus*	SIO‐BIC E6460	PZ015807	PZ091738	10‐Apr‐2013	Strait of Magellan	SoM	−53.509117	−70.406417
*Labidiaster radiosus*	SIO‐BIC E6465	PZ015808	PZ091739	10‐Apr‐2013	Strait of Magellan	SoM	−53.509117	−70.406417
*Labidiaster radiosus*	SIO‐BIC E6513	PZ015809	PZ091740	10‐Apr‐2013	Strait of Magellan	SoM	−53.509117	−70.406417
*Labidiaster radiosus*	SIO‐BIC E6505	PZ015810	—	28‐Apr‐2013	Falkland Islands/Islas Malvinas	FI	−51.637767	−57.4938
*Labidiaster radiosus*	SIO‐BIC E6506	PZ015811	PZ091741	28‐Apr‐2013	Falkland Islands/Islas Malvinas	FI	−51.637767	−57.4938
*Labidiaster radiosus*	SIO‐BIC E11585	PZ015812	PZ091742	14‐Oct‐2011	Discovery Bank	DB	−60.15318	−34.87701
*Labidiaster radiosus*	SIO‐BIC W1683	PZ015813	PZ091743	13‐Oct‐2011	Discovery Bank	DB	−60.11104	−34.82669
*Labidiaster radiosus*	SIO‐BIC E5044	PZ015814	PZ091744	14‐Oct‐2011	Discovery Bank	DB	−60.12372	−34.93466
*Labidiaster radiosus*	SIO‐BIC E5051	PZ015815	PZ091745	10‐Oct‐2011	Herdman Bank	HB	−59.86338	−32.47014
*Labidiaster radiosus*	SIO‐BIC E6418	PZ015816	PZ091746	28‐Apr‐2013	Falkland Islands/Islas Malvinas	FI	−51.637767	−57.4938
*Labidiaster radiosus*	SIO‐BIC E6419	PZ015817	PZ091747	28‐Apr‐2013	Falkland Islands/Islas Malvinas	FI	−51.637767	−57.4938
*Labidiaster radiosus*	SIO‐BIC E6420	PZ015818	PZ091748	28‐Apr‐2013	Falkland Islands/Islas Malvinas	FI	−51.637767	−57.4938
*Labidiaster radiosus*	SIO‐BIC E6422	PZ015819	PZ091749	28‐Apr‐2013	Falkland Islands/Islas Malvinas	FI	−51.637767	−57.4938
*Labidiaster radiosus*	SIO‐BIC E6436	PZ015820	PZ091750	28‐Apr‐2013	Falkland Islands/Islas Malvinas	FI	−51.637767	−57.4938
*Labidiaster radiosus*	SIO‐BIC E6437	PZ015821	PZ091751	28‐Apr‐2013	Falkland Islands/Islas Malvinas	FI	−51.637767	−57.4938
*Labidiaster radiosus*	SIO‐BIC E6438	PZ015822	PZ091752	28‐Apr‐2013	Falkland Islands/Islas Malvinas	FI	−51.637767	−57.4938
*Labidiaster radiosus*	SIO‐BIC E6439	PZ015823	PZ091753	28‐Apr‐2013	Falkland Islands/Islas Malvinas	FI	−51.637767	−57.4938
*Labidiaster radiosus*	SIO‐BIC E6440	PZ015824	PZ091754	28‐Apr‐2013	Falkland Islands/Islas Malvinas	FI	−51.637767	−57.4938
*Labidiaster radiosus*	SIO‐BIC E6448	PZ015825	PZ091755	10‐Apr‐2013	Strait of Magellan	SoM	−53.509117	−70.406417
*Labidiaster radiosus*	SIO‐BIC E6461	PZ015826	PZ091756	10‐Apr‐2013	Strait of Magellan	SoM	−53.509117	−70.406417
*Labidiaster radiosus*	SIO‐BIC E6463	PZ015827	PZ091757	10‐Apr‐2013	Strait of Magellan	SoM	−53.509117	−70.406417
*Labidiaster radiosus*	SIO‐BIC E6473	PZ015828	PZ091758	10‐Apr‐2013	Strait of Magellan	SoM	−53.509117	−70.406417
*Labidiaster radiosus*	SIO‐BIC E6474	PZ015829	PZ091759	10‐Apr‐2013	Strait of Magellan	SoM	−53.509117	−70.406417
*Labidiaster radiosus*	SIO‐BIC E6475	PZ015830	PZ091760	10‐Apr‐2013	Strait of Magellan	SoM	−53.509117	−70.406417
*Labidiaster radiosus*	SIO‐BIC E6476	PZ015831	PZ091761	28‐Apr‐2013	Falkland Islands/Islas Malvinas	FI	−51.637767	−57.4938
*Labidiaster radiosus*	SIO‐BIC E6477	PZ015832	PZ091762	28‐Apr‐2013	Falkland Islands/Islas Malvinas	FI	−51.637767	−57.4938
*Labidiaster radiosus*	SIO‐BIC E6480	PZ015833	PZ091763	10‐Apr‐2013	Strait of Magellan	SoM	−53.509117	−70.406417
*Labidiaster radiosus*	SIO‐BIC E6481	PZ015834	PZ091764	10‐Apr‐2013	Strait of Magellan	SoM	−53.509117	−70.406417
*Labidiaster radiosus*	SIO‐BIC E6482	PZ015835	PZ091765	10‐Apr‐2013	Strait of Magellan	SoM	−53.509117	−70.406417
*Labidiaster radiosus*	SIO‐BIC E6483	PZ015836	PZ091766	10‐Apr‐2013	Strait of Magellan	SoM	−53.509117	−70.406417
*Labidiaster radiosus*	SIO‐BIC E6484	PZ015837	PZ091767	10‐Apr‐2013	Strait of Magellan	SoM	−53.509117	−70.406417
*Labidiaster radiosus*	SIO‐BIC E6485	PZ015838	PZ091768	10‐Apr‐2013	Strait of Magellan	SoM	−53.509117	−70.406417
*Labidiaster radiosus*	SIO‐BIC E6486	PZ015839	PZ091769	10‐Apr‐2013	Strait of Magellan	SoM	−53.509117	−70.406417
*Labidiaster radiosus*	SIO‐BIC E6496	PZ015840	PZ091770	10‐Apr‐2013	Strait of Magellan	SoM	−53.509117	−70.406417
*Labidiaster radiosus*	SIO‐BIC E6507	PZ015841	PZ091771	28‐Apr‐2013	Falkland Islands/Islas Malvinas	FI	−51.637767	−57.4938
*Labidiaster radiosus*	SIO‐BIC E6508	PZ015842	PZ091772	28‐Apr‐2013	Falkland Islands/Islas Malvinas	FI	−51.637767	−57.4938
*Labidiaster radiosus*	SIO‐BIC E6509	PZ015843	PZ091773	28‐Apr‐2013	Falkland Islands/Islas Malvinas	FI	−51.637767	−57.4938
*Labidiaster radiosus*	SIO‐BIC E6510	PZ015844	—	28‐Apr‐2013	Falkland Islands/Islas Malvinas	FI	−51.637767	−57.4938
*Labidiaster radiosus*	SIO‐BIC E6511	PZ015845	PZ091774	28‐Apr‐2013	Falkland Islands/Islas Malvinas	FI	−51.637767	−57.4938
*Labidiaster radiosus*	SIO‐BIC E6512	PZ015846	PZ091775	28‐Apr‐2013	Falkland Islands/Islas Malvinas	FI	−51.637767	−57.4938
*Labidiaster radiosus*	SIO‐BIC E6516	PZ015847	PZ091776	28‐Apr‐2013	Falkland Islands/Islas Malvinas	FI	−51.637767	−57.4938
*Labidiaster radiosus*	SIO‐BIC E6518	PZ015848	PZ091777	28‐Apr‐2013	Falkland Islands/Islas Malvinas	FI	−51.637767	−57.4938
*Labidiaster radiosus*	SIO‐BIC W1239	PZ015849	—	01‐Jun‐2008	Heard Island: Evitas Patch	HI	−52.77	74.65167
*Labidiaster radiosus*	SIO‐BIC W1263	PZ015850	—	04‐May‐2005	Heard Island	HI	−53.10367	72.905
*Labidiaster radiosus*	SIO‐BIC W1353	PZ015851	—	01‐Jun‐2008	Heard Island: Evitas Patch	HI	−52.79333	74.70167
*Labidiaster radiosus*	SIO‐BIC W1354	PZ015852	—	01‐Jun‐2008	Heard Island: Evitas Patch	HI	−52.79333	74.70167
*Labidiaster radiosus*	SIO‐BIC W1355	PZ015853	—	01‐Jun‐2008	Heard Island: Evitas Patch	HI	−52.79333	74.70167
*Labidiaster radiosus*	SIO‐BIC W1371	PZ015854	—	01‐Jun‐2008	Heard Island: Evitas Patch	HI	−52.91	74.49167
*Labidiaster radiosus*	SIO‐BIC W1372	PZ015855	—	01‐Jun‐2008	Heard Island: Evitas Patch	HI	−52.91	74.49167
*Labidiaster radiosus*	SIO‐BIC W1373	PZ015856	—	01‐Jun‐2008	Heard Island: Evitas Patch	HI	−52.97833	74.755
*Labidiaster radiosus*	SIO‐BIC W1374	PZ015857	—	01‐Jun‐2008	Heard Island: Evitas Patch	HI	−52.97833	74.755
*Labidiaster radiosus*	SIO‐BIC W1378	PZ015858	—	01‐Jun‐2008	Heard Island: Evitas Patch	HI	−52.80167	74.63833
*Labidiaster radiosus*	WAM Z44048	PZ015859	PZ091778	01‐Mar‐2017	Leclaire Rise	LR	−49.7335	64.8939

**TABLE A3:** Showing results for partial mantel test statistic (*r*) with statistical significance indicated by an asterisk (*) for a *p*‐value < 0.001. The partial mantel test for COI and intron 7 for both *Labidiaster annulatus* and *Labidiaster radiosus* shows the relationship between the genetic distance and geographical distance (km) when depth is kept constant, and the relationship between the genetic distance and depth when geographical distance (km) is kept constant.

	Genetic distance and geographical distance (depth = constant)		Genetic distance and depth (geographic distance = constant)	
	Mantel statistic *r*	Significance	Mantel statistic *r*	Significance
***Labidiaster annulatus* (COI)**				
Pearson	−0.1089485	0.9958	0.02295422	0.3021
Spearman	−0.8003983	0.9955	0.02663327	0.2034
Kendall	−0.0600379	1	0.01167774	0.3
***Labidiaster radiosus* (COI)**				
Pearson	0.404819	0.0001*	0.5321073	0.0001*
Spearman	0.3515359	0.0001*	0.584597	0.0001*
Kendall	0.2609783	0.0001*	0.4664297	0.0001*
***Labidiaster radiosus* Southern Ocean subclade 1 (COI)**				
Pearson	0.2847502	0.0001*	0.08967466	0.1094
Spearman	0.1466662	0.0017	0.1024561	0.058
Kendall	0.1126585	0.0031	0.0764449	0.059
***Labidiaster radiosus* Southern South America subclade 2 (COI)**				
Pearson	0.03225845	0.1853	−0.0565044	0.8109
Spearman	0.04723018	0.1856	−0.0535975	0.8134
Kendall	0.03217577	0.1807	−0.0405758	0.822
***Labidiaster annulatus* (Intron 7)**				
Pearson	−0.0043643	0.5718	0.07995928	0.019
Spearman	0.01482813	0.0665	0.00770442	0.1879
Kendall	0.01234975	0.09	0.00774046	0.12
***Labidiaster radiosus* (Intron 7)**				
Pearson	0.244135	0.0001*	0.2361276	0.0001*
Spearman	0.2108377	0.0001*	0.223451	0.0001*
Kendall	0.1775902	0.01	0.1852581	0.01
