# Supplementary material for: Contrasting Dispersal Patterns of Co‐Occurring Benthic Sister Sea Star Labidiaster Species (Asteroidea: Heliasteridae) in the Southern Ocean
Source: Ecol Evol. 2026 Apr 10;16(4):e73450. doi: 10.1002/ece3.73450 (PMC13068501; doi:10.1002/ece3.73450)
Supplement: Supplementary file 1 — Figure S1: ece373450‐sup‐0001‐Supinfo.docx. Labidiaster radiosus early bipinnaria larva at 100× magnification (Mary Sewell, pers. comm.). Collected at Cape Hallett, Ross Sea on 3 December 2004 (GenBank: GU227094). Figure S2: Maximum clade credibility tree showing median divergence time estimates on nodes for Labidiaster species as estimated from COI sequences. Dashed boxes show Labidiaster annulatus (Clade I) and Labidiaster radiosus (Clade II), and subclades within Labidiaster radiosus (Clade II) are labelled respectively. Purple boxes on node bars represent 95% height HPD. The scale bar represents branch lengths, and the scale axis indicates million years before present. Figure S3: Extended Coalescent Bayesian skyline plot for Labidiaster annulatus showing effective population sizes (log(Ne)) across time (thousand years ago) for combined COI and Intron 7. The dashed black line represents the median and the grey shading on either side of the line represents 95% highest posterior density. Figure S4: Extended Coalescent Bayesian skyline plot for Labidiaster radiosus showing effective population sizes (log(Ne)) across time (thousand years ago) for combined COI and Intron 7. The dashed black line represents the median and the grey shading on either side of the line represents 95% highest posterior density. Figure S5: Relationship between genetic distance, represented by the pairwise genetic differentiation (FST) and the linearized FST (FST/(1‐FST)), and the geographical distances (km) among locations for Labidiaster annulatus COI (a, c) and intron 7 (b, d). The black line represents the linear regression line with its standard error shown by the grey shading above and below the black line. The linear equation, the coefficient of determination (r2) and its associated significance (p‐value) is shown in the top right of the plot. The p‐value is statistically significant at p < 0.05 and indicated by an asterisk (*). Locations for COI with n < 3 (Burdwood Bank, Falkland Isl [file ECE3-16-e73450-s001.docx]

**SUPPORTING INFORMATION**

**
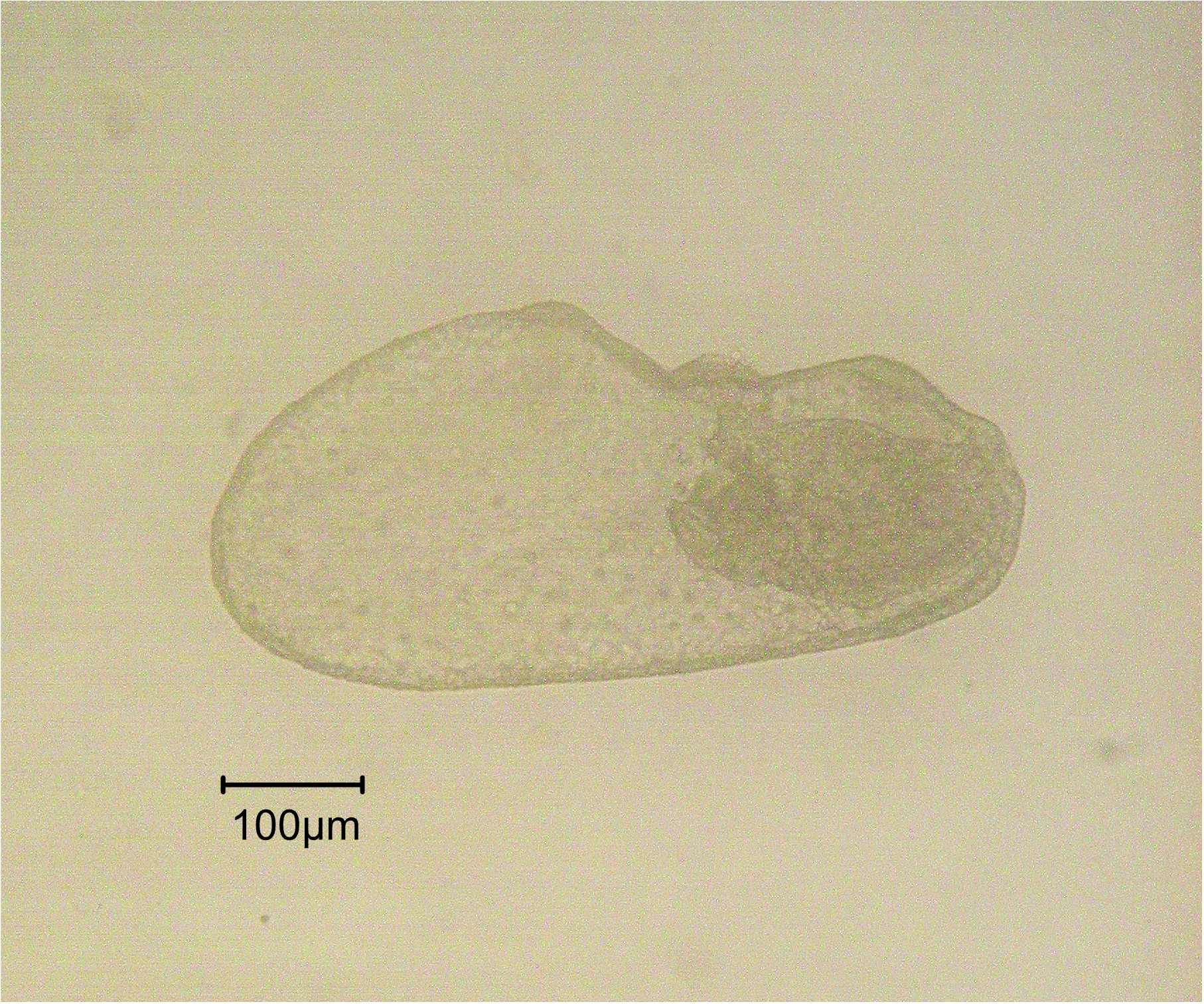
**

Figure S1: *Labidiaster radiosus* early bipinnaria larva at 100x magnification (Mary Sewell, pers. comm.). Collected at Cape Hallett, Ross Sea on 3 December 2004 (GenBank: GU227094).

Figure S2: Maximum clade credibility tree showing median divergence time estimates on nodes for *Labidiaster* species as estimated from COI sequences. Dashed boxes show *Labidiaster* *annulatus* (Clade I) and *Labidiaster radiosus* (Clade II), and subclades within *Labidiaster radiosus* (Clade II) are labelled respectively. Purple boxes on node bars represent 95% height HPD. The scale bar represents branch lengths, and the scale axis indicates million years before present.


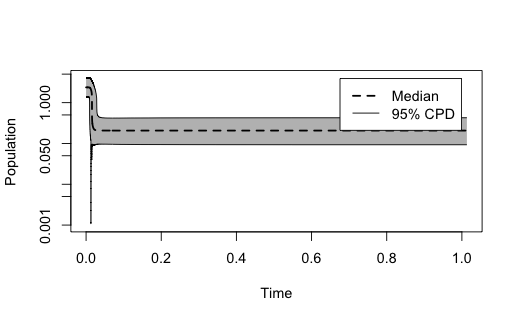


Figure S3: Extended Coalescent Bayesian skyline plot for *Labidiaster annulatus* showing effective population sizes (log(Ne)) across time (thousand years ago) for combined COI and Intron 7. The dashed black line represents the median and the grey shading on either side of the line represents 95% highest posterior density.


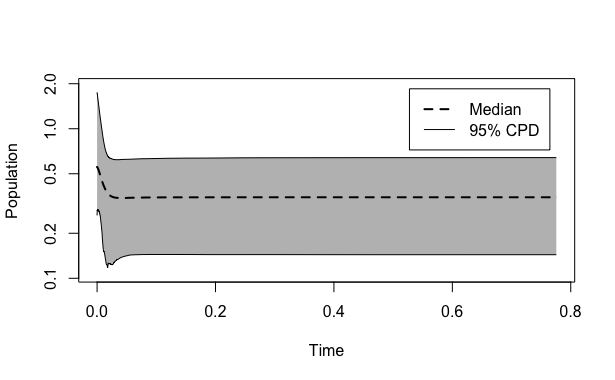


Figure S4: Extended Coalescent Bayesian skyline plot for *Labidiaster radiosus* showing effective population sizes (log(Ne)) across time (thousand years ago) for combined COI and Intron 7. The dashed black line represents the median and the grey shading on either side of the line represents 95% highest posterior density.


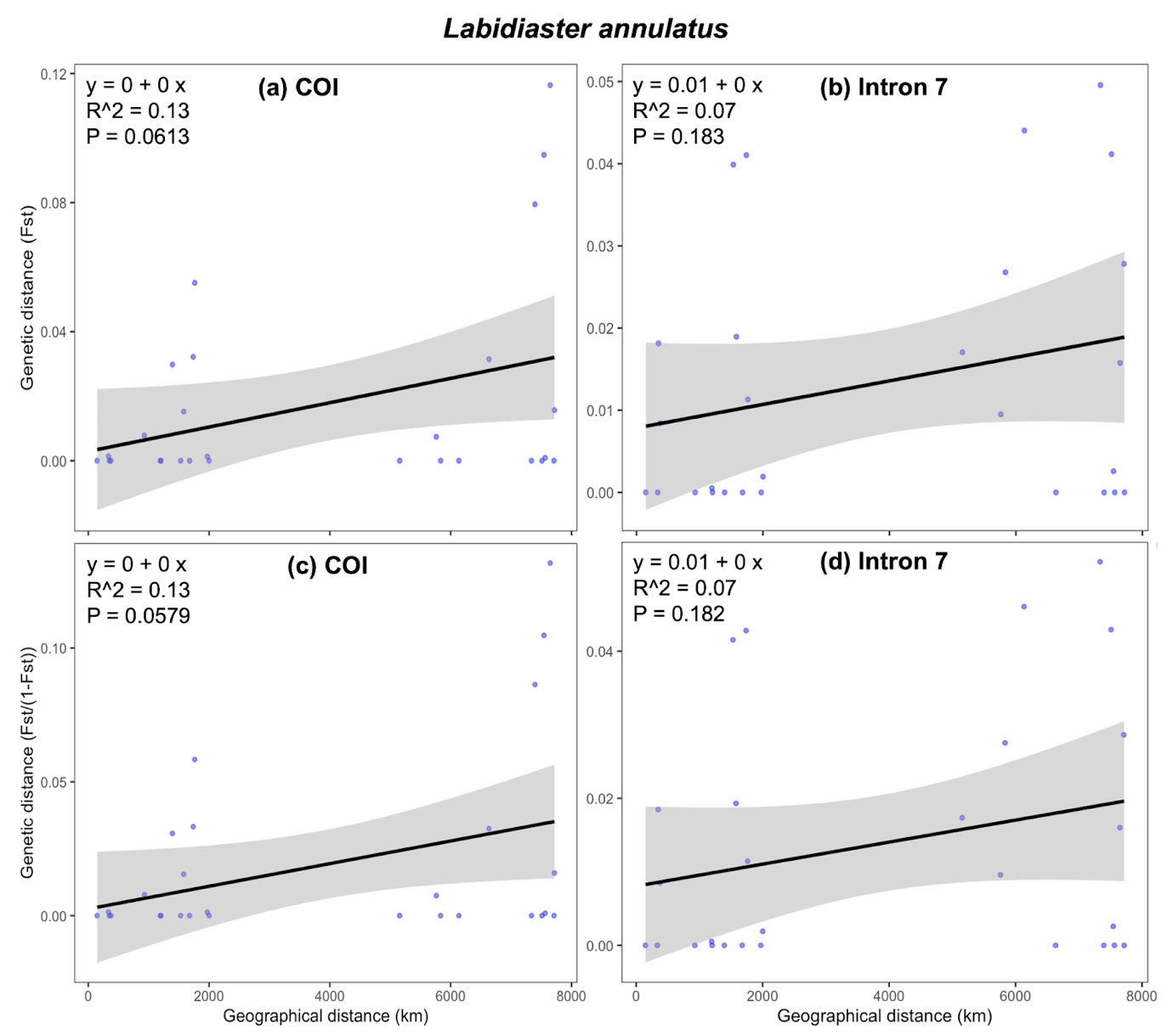


Figure S5: The relationship between genetic distance, represented by the pairwise genetic differentiation (F_ST_) and the linearised F_ST_ (F_ST_/(1-F_ST_)), and the geographical distances (km) among locations for *Labidiaster annulatus* COI (a, c) and intron 7 (b, d). The black line represents the linear regression line with its standard error shown by the grey shading above and below the black line. The linear equation, the coefficient of determination (r^2^) and its associated significance (*p*-value) is shown in the top right of the plot. The *p*-value is statistically significant at *p* < 0.05 and indicated by an asterisk (*). Locations for COI with n < 3 (Burdwood Bank, Falkland Islands/Malvinas East, South Orkney Islands, Admiralty Seamount, Scott Island) and for intron 7 with n < 6 (Burdwood Bank, Falkland Islands/Malvinas East, South Orkney Islands, Scott Island) were not used in the plot.


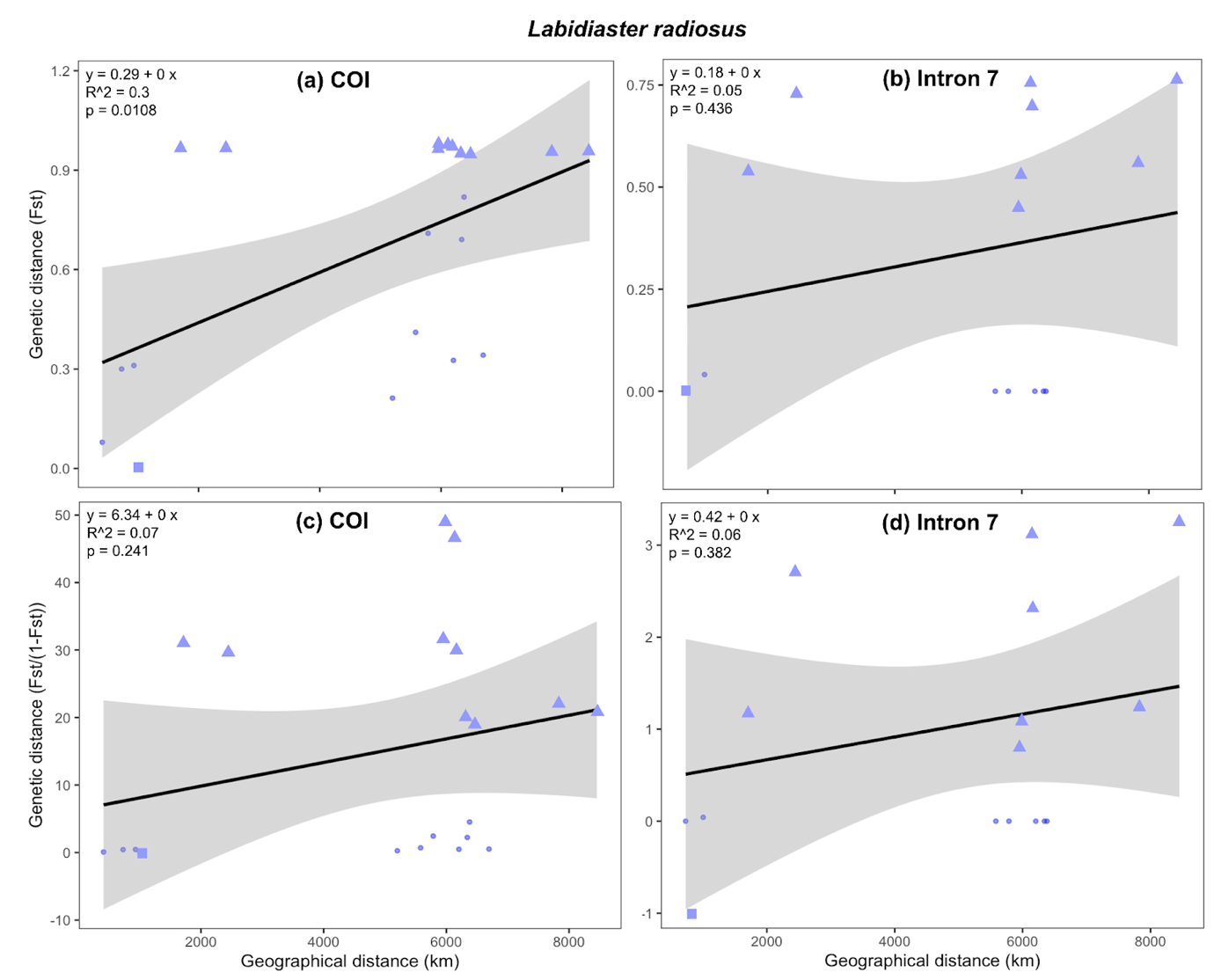


Figure S6: The relationship between genetic distance, represented by the pairwise genetic differentiation (F_ST_) and the linearised F_ST_ (F_ST_/(1-F_ST_)), and the geographical distances (km) among locations for *Labidiaster radiosus* COI (a, c) and intron 7 (b, d). The black line represents the linear regression line with its standard error shown by the grey shading above and below the black line. The linear equation, the coefficient of determination (R^2) and its associated significance (*p*-value) is shown in the top right of the plot. The *p*-value is statistically significant at *p* < 0.05 and indicated by an asterisk (*). Locations for COI with n < 3 (Herdman Bank, Leclaire Rise) and for intron 7 with n < 6 (Herdman Bank, Leclaire Rise, Admiralty Seamount) were not used in the plot. Square indicates among locations north of the Antarctic Polar Front, triangles indicate between locations north and south of the Antarctic Polar Front, and circles indicate only among locations south of the Antarctic Polar Front.

Table S1: AMOVA results for *L. annulatus* locations. Locations within the sequence dataset COI with n < 3 (Burdwood Bank, Falkland Islands/Malvinas East, South Orkney Islands, Admiralty Seamount, Scott Island) and intron 7 with n < 6 (Burdwood Bank, Falkland Islands/Malvinas East, South Orkney Islands, Scott Island) were not included in the analysis. *Labidiaster radiosus* locations for COI with n < 3 (Herdman Bank, Leclaire Rise) and for intron 7 with n < 6 (Herdman Bank, Leclaire Rise, Admiralty Seamount) were not included in the AMOVA analysis. Significant *p*-value (*p* < 0.05) is shown with an asterisk (*).

|  | Sum of squares | df | Variance components | Percentage of variation | F_ST_ | *p*-value |
| --- | --- | --- | --- | --- | --- | --- |
| *Labidiaster annulatus* | | | | | | |
| **COI** |  |  |  |  |  |  |
| Among locations | 2.08 | 7 | 0.00199 | 0.84919 | 0.00849 | 0.1261 |
| Within locations | 60.498 | 260 | 0.23268 | 99.15081 |  |  |
| Total | 62.578 | 267 | 0.23468 |  |  |  |
| **Intron7** |  |  |  |  |  |  |
| Among locations | 3.351 | 7 | 0.00123 | 0.30175 | 0.00302 | 0.28152 |
| Within locations | 197.525 | 486 | 0.40643 | 99.69825 |  |  |
| Total | 200.877 | 493 | 0.40766 |  |  |  |
| *Labidiaster radiosus* | | | | | | |
| **COI** |  |  |  |  |  |  |
| Among locations | 148.89 | 6 | 2.05445 | 92.38166 | 0.99396 | 0* |
| Within locations | 13.723 | 81 | 0.16942 | 7.61834 |  |  |
| Total | 162.614 | 87 | 2.22387 |  |  |  |
| **Intron7** |  |  |  |  |  |  |
| Among locations | 82.08 | 5 | 0.72923 | 51.08392 | 0.51084 | 0* |
| Within locations | 92.174 | 132 | 0.69829 | 48.91608 |  |  |
| Total | 174.254 | 137 | 1.42752 |  |  |  |

Table S2: Migrate-n results for *Labidiaster annulatus* (a) showing location groupings used and (b) models implemented in migrate-n ranking from highest to lowest probability calculated from BEZIER log(mL).

| **(a)** | **Location** | **Location Number** | **Gene** | **Number of Sequences** |  |  |  |  |  |  |  |
| --- | --- | --- | --- | --- | --- | --- | --- | --- | --- | --- | --- |
|  | Shag Rocks | 1 | COI | 54 |  |  |  |  |  |  |  |
|  | South Georgia West | 2 | COI | 20 |  |  |  |  |  |  |  |
|  | South Georgia East | 3 | COI | 23 |  |  |  |  |  |  |  |
|  | Visokoi Island (South Sandwich Island) | 4 | COI | 22 |  |  |  |  |  |  |  |
|  | Candlemas Island (South Sandwich Island) | 5 | COI | 16 |  |  |  |  |  |  |  |
|  | Montagu Island (South Sandwich Island) | 6 | COI | 18 |  |  |  |  |  |  |  |
|  | Elephant Island | 7 | COI | 21 |  |  |  |  |  |  |  |
|  | South Shetland Islands | 8 | COI | 14 |  |  |  |  |  |  |  |
|  | Bransfield Strait Mouth | 9 | COI | 21 |  |  |  |  |  |  |  |
|  | Bransfield Strait | 10 | COI | 17 |  |  |  |  |  |  |  |
|  | Total |  | COI | 226 |  |  |  |  |  |  |  |
| **(b)** | **Model** | **BEZIER log(mL)** | **LBF** | **Probability** | **Rank** |  |  |  |  |  |  |
|  | full migration | -1856 | -9.7 | -9.70 | 2 |  |  |  |  |  |  |
|  | panmixia | -1846 | 0 | 0.00 | 1 |  |  |  |  |  |  |
|  | Stepping-stone N→S | -1995 | -149.1 | -149.09 | 3 |  |  |  |  |  |  |
|  | Stepping-stone S→N | -2009 | -162.69 | -162.68 | 4 |  |  |  |  |  |  |
|  |  |  |  |  |  |  |  |  |  |  |  |

Table S3: Migrate-n results for *Labidiaster radiosus* (a) showing location groupings used and (b) models implemented in migrate-n ranking from highest to lowest probability calculated from BEZIER log(mL).

| **(a)** | **Location** | **Location Number** | **Gene** | **Number of Sequences** |  |  |
| --- | --- | --- | --- | --- | --- | --- |
|  | Strait of Magellan | 1 | COI | 20 |  |  |
|  | Falkland Islands/Malvinas | 2 | COI | 21 |  |  |
|  | Discovery Bank | 3 | COI | 16 |  |  |
|  | Heard Island | 4 | COI | 17 |  |  |
|  | Ross Sea region (including Admiralty Seamount and Scott Island) | 5 | COI | 16 |  |  |
|  | Total |  | COI | 90 |  |  |
| **(b)** | **Model** | **BEZIER log(mL)** | **LBF** | **Probability** | **Rank** |  |
|  | Panmixia | -1463.45 | 0 | 1 | 1 |  |
|  | Full migration | -1489.08 | -25.63 | 7.4E-12 | 2 |  |
|  | Stepping-stone | -1519.78 | -56.33 | 3.4371E-25 | 3 |  |
|  | Unidirectional anti-clockwise | -1535.81 | -72.36 | 3.8E-32 | 4 |  |
|  | Unidirectional clockwise | -1542.93 | -79.48 | 3.0E-35 | 5 |  |
